# Photodynamic Therapy Combined with Anticancer Drug Therapy in the Treatment of Malignant Neoplasms

**DOI:** 10.3390/cells15090781

**Published:** 2026-04-25

**Authors:** Igor Reshetov, Anna Alyasova, Olga Shpileva, Pavel Karalkin, Kanamat Efendiev, Daria Pominova, Victor Loschenov, Dinara Ilyasova, Yulia Agakina, Aida Gilyadova, Vadim Cheremisov, Andrey Stetsiuk, Alena Mamedova, Arina Petrova, Polina Kozlova, Ekaterina Rostislavova, Valeria Sudarkina, David Abadzhyan, Artem Shiryaev

**Affiliations:** 1 Department of Oncology, Radiotherapy and Reconstructive Surgery, Levshin Institute of Cluster Oncology, Sechenov First Moscow State Medical University, 119435 Moscow, Russiapetrova_a_a@staff.sechenov.ru (A.P.);; 2 Prokhorov General Physics Institute of the Russian Academy of Sciences, 119991 Moscow, Russia; 3 Department of Laser Micro-, Nano-, and Biotechnology, Institute of Engineering Physics for Biomedicine, National Research Nuclear University “MEPhI”, 115409 Moscow, Russia; 4 Institute of Mathematics and Natural Sciences, Kabardino-Balkarian State University, 360004 Nalchik, Russia

**Keywords:** photodynamic therapy, photosensitizers, anticancer chemotherapy, chemoresistance, immune checkpoint inhibitors, anticancer vaccines, nanoparticles, immunoconjugates

## Abstract

**Highlights:**

**What are the main findings?**

- Photodynamic therapy (PDT) demonstrates strong synergy with chemotherapy by enhancing drug delivery and overcoming multi-drug resistance.
- The combination of PDT and immunotherapy triggers immunogenic cell death, converting immunologically “cold” tumors into “hot” ones.

**What are the implications of the main findings?**

- Clinical trials demonstrate promising outcomes for combined PDT-drug regimens in treating cancers.
- Personalized treatment protocols and advanced dosimetry are essential for the clinical translation of combined PDT therapies.

**Abstract:**

Background: Photodynamic therapy (PDT) has emerged as a powerful minimally invasive modality for cancer treatment. However, its efficacy as a monotherapy is often limited by oxygen dependence and limited light penetration. Combining PDT with systemic anticancer drug therapies offers a promising strategy to achieve synergistic effects and overcome resistance. Objective: This review aims to provide a systematic analysis of the mechanisms and clinical potential of combining PDT with chemotherapy, targeted therapy, and immunotherapy, focusing on recent advancements and nanotechnology-based delivery systems. Methods: A comprehensive literature search was performed using PubMed and Scopus databases. The analysis focused on peer-reviewed studies published over the last 10 years addressing synergistic molecular pathways, co-delivery nanoplatforms, and clinical trial outcomes. Results: The combination of PDT with chemotherapy enhances drug accumulation via vascular photosensitization and can overcome multi-drug resistance. Integration with immunotherapy, particularly immune checkpoint inhibitors and tumor vaccines, triggers immunogenic cell death (ICD), leading to systemic antitumor responses. Nanotechnology provides a versatile platform for the targeted co-delivery of photosensitizers and pharmacological agents, significantly reducing systemic toxicity. Conclusions: Combined PDT–drug regimens demonstrate superior therapeutic efficacy compared to monotherapies. Future clinical translation requires the standardization of dosimetry and the development of multifunctional nanomedicines to enable personalized treatment protocols.

## 1. Introduction

According to current projections, by 2030, more than 13.1 million people worldwide may die because of incurable malignant neoplasms, making malignancies the second leading cause of death after cardiovascular diseases [1]. The treatment of malignant neoplasms remains a major global healthcare challenge, requiring the development of novel and more effective therapeutic strategies for various types of cancer [2]. While modern oncology has led to substantial advances in developing targeted and immunotherapeutic approaches, the complete eradication of tumors remains difficult due to the development of multidrug resistance and inherent tumor heterogeneity. One such promising strategy is photodynamic therapy (PDT), particularly due to its potential synergistic interaction with conventional treatment modalities, including chemotherapy, immunotherapy, and targeted agents, as well as surgical intervention and radiotherapy [3]. Recent studies in the field indicate that photodynamic therapy is evolving from a conventional treatment into a sophisticated bioengineering platform. The integration of precision imaging, controlled priming of the tumor microenvironment (TME), and advanced dosimetry is crucial for enhancing therapeutic outcomes [4]. Furthermore, the role of PDT is no longer limited to local cytotoxicity; it is increasingly recognized as a potent trigger for immunogenic cell death (ICD), paving the way for synergistic combinations with immunotherapy [5]. PDT relies on the interaction of three key components: a photosensitizer (PS) that accumulates in the target tissue, the light of a specific wavelength, and molecular oxygen. Upon activation, photochemical reactions (primarily Type II) generate reactive oxygen species (ROS), specifically singlet oxygen (^1^O_2_), which induce direct tumor cell death, destruction of the tumor microvasculature, and the initiation of a robust systemic immune response. Despite its high selectivity and minimal systemic toxicity, the efficacy of PDT as a monotherapy is often constrained by several physiological barriers. These include the limited penetration depth of light into biological tissues, the dependence of therapeutic outcomes on local oxygenation levels (which is critical in the context of tumor hypoxia), and the challenges of achieving sufficient PS concentrations in deep-seated metastatic lesions. Conversely, traditional systemic therapies, such as chemotherapy, are frequently limited by severe side effects and the rapid emergence of resistance. The rationale for combining PDT with anticancer drug therapy lies in several unique synergistic mechanisms. First, PDT can induce vascular priming, temporarily increase the permeability of the tumor vasculature and enhance the accumulation of subsequent chemotherapeutic agents via the enhanced permeability and retention (EPR) effect [4]. Second, photodynamic action can effectively overcome multidrug resistance by inactivating efflux pump proteins, such as P-glycoprotein, thereby restoring the sensitivity of resistant cells to drugs like cisplatin or doxorubicin [6]. Third, PDT triggers immunological synergy by inducing ICD [7] and the release of damage-associated molecular patterns (DAMPs) [8]. This process “primes” the immune system, potentially converting “cold” non-responsive tumors into “hot” tumors that are more susceptible to immune checkpoint inhibitors and vaccines. Furthermore, nanotechnology serves as a critical bridge in these combination protocols. The development of multifunctional nanocarriers allows for the targeted co-delivery of PSs and pharmacological agents, ensuring synchronized release at the tumor site while minimizing off-target effects.

While several prior reviews have addressed individual aspects of PDT combination strategies in isolation—including PDT combined with chemotherapy [7,9], PDT combined with immune checkpoint inhibitors [10], and nanotechnology-based PDT delivery systems [11]—the present work is distinguished by its integrative and mechanistically grounded scope, systematically linking PDT mechanisms to specific combination rationales and clinical outcomes across all major modalities—chemotherapy, immunotherapy, targeted therapy, and nanotechnology-based co-delivery platforms—within a single unified framework. Uniquely, this review incorporates a critical appraisal of the translational gap between preclinical and clinical settings, including an analysis of dosimetry limitations [12,13], hypoxia-related barriers [14,15], and regulatory challenges [16]. Furthermore, the manuscript integrates data from recently published studies, including the most recent Phase III clinical trial data, and addresses clinically relevant topics such as interstitial PDT and patient safety protocols that are rarely covered in combination therapy reviews. The aim of the present review is therefore to provide a comprehensive and clinically actionable synthesis of the current evidence on combining PDT with anticancer pharmacotherapy, highlighting the most promising strategies and the key steps required for their implementation in routine oncological practice. Unlike previous reviews, this work integrates mechanistic, clinical, and translational perspectives within a unified framework.

To our knowledge, no existing review simultaneously addresses all four major PDT combination modalities—chemotherapy, immunotherapy (including immune checkpoint inhibitors (ICIs) and dendritic cell (DC) vaccines), targeted therapy (photoimmunoconjugates and near-infrared photoimmunotherapy (NIR-PIT)), and nanotechnology co-delivery platforms—within a single comparative framework that links photophysical mechanisms to clinical outcomes. While authoritative reviews have addressed PDT-immunotherapy combinations [5] or the engineering principles of PDT [4] in depth, they often overlook chemotherapy-specific resistance mechanisms, cyclooxygenase-2 (COX-2)-mediated immunosuppression, or the translational gap in nanoplatform manufacturing. Additionally, the present review uniquely incorporates a critical ‘lessons learned’ and ‘What Not To Do’ analysis—a perspective absent from the existing literature—and addresses interstitial PDT and patient safety protocols that are rarely discussed in the context of combination therapy.

## 2. Materials and Methods

A literature review on PDT was conducted using the Scopus and PubMed. From several thousand studies on the use of PDT for malignant tumors, studies investigating the mechanisms of PDT, as well as those addressing the use of PDT in combination with anticancer agents, were selected. The search included the following keywords: photodynamic therapy, photosensitizers, anticancer chemotherapy, chemoresistance, immune checkpoint inhibitors, anticancer vaccines, nanoparticles, immunoconjugates.

## 3. Principles of PDT

### 3.1. Mechanisms of PDT

**Figure 1:**
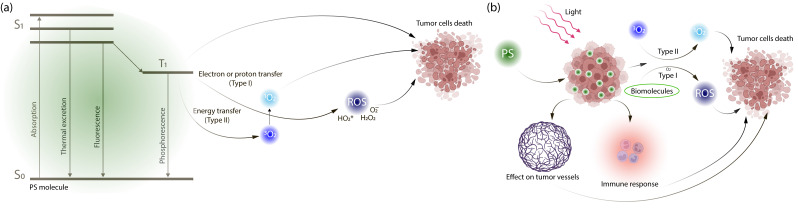
Photophysical basis and antitumor mechanisms of PDT. (**a**) Jablonski diagram illustrating PS photoexcitation and the two principal ROS-generating pathways. Following intersystem crossing to the triplet state (T_1_), the PS engages either the Type II pathway—energy transfer to ground-state oxygen (^3^O_2_) yielding singlet oxygen (^1^O_2_)—or the Type I pathway—electron transfer to biological substrates yielding ROS. The efficiency of each pathway is governed by the triplet quantum yield (Φ_T_), triplet-state lifetime (τ_T_), and ^1^O_2_ quantum yield (Φ_Δ_). (**b**) Biological consequences of ROS generation at the tumor level. PSs selectively accumulate in tumor tissue and, upon light activation, initiate three complementary antitumor mechanisms: direct tumor cell cytotoxicity, vascular disruption through ROS-mediated endothelial damage, and systemic immune activation via ICD and DAMPs release. Note: PS—photosensitizer; ROS—reactive oxygen species; S_0_/S_1_/T_1_—ground, first excited singlet, and triplet states; ^3^O_2_/^1^O_2_—ground-state and singlet oxygen; O_2_^−^—superoxide; HO_2_*—hydroperoxyl radical; H_2_O_2_—hydrogen peroxide; Φ_T_—triplet quantum yield; τ_T_—triplet-state lifetime; Φ_Δ_—singlet oxygen quantum yield; ICD—immunogenic cell death; DAMPs—damage-associated molecular patterns.

PDT is a therapeutic modality used in the treatment of various diseases that exploits the photophysical and chemical properties of PSs [9]. The main advantages of PDT include its noninvasive nature, the absence of complications associated with surgical intervention, and the minimization of side effects typical of chemotherapy and radiotherapy. The unique mechanism of PDT is attributed to the use of photosensitizing agents that can preferentially accumulate in tumor cells following systemic or local administration [17]. Upon light irradiation at a specific wavelength, PSs transition from the ground state to an excited state [18] and, in the presence of molecular oxygen, generates ROS through type I and type II photochemical reactions [19]. ROS generation efficiency is primarily governed by the photophysical properties of the PS, specifically the quantum yield of the triplet state (Φ_T_) and the triplet lifetime (τ_T_). A high Φ_T_ ensures that a significant fraction of excited singlet states (S_1_) undergoes intersystem crossing to the long-lived triplet state (T_1_), which is critical for interaction with molecular oxygen or endogenous substrates before radiative or non-radiative decay occurs. In Type II pathways, the energy transfer efficiency to ground-state oxygen (^3^O_2_) to form singlet oxygen (^1^O_2_) is defined by the ^1^O_2_ quantum yield (Φ_Δ_) (Figure 1a). Conversely, Type I pathways involve electron transfer from T_1_ to biological substrates, generating radical species like superoxide (O_2_^−^), the hydroperoxyl radical (HO_2_*), and hydrogen peroxide (H_2_O_2_). Recognizing the limitations of oxygen-dependent Type II processes, modern research increasingly focuses on shifting this kinetics toward Type I or oxygen-independent mechanisms (e.g., photoactivated chemotherapy (PACT)) to ensure therapeutic efficacy in the severely hypoxic core of solid tumors, where ^3^O_2_ concentrations drop below the threshold required for efficient Type II PDT. The main mechanisms of the PDT action are illustrated in Figure 1b.

As illustrated in Figure 1b, the three antitumor mechanisms of PDT—direct cytotoxicity, vascular disruption, and immune activation—are not independent but mutually reinforcing: ROS-mediated vascular damage exacerbates tumor hypoxia and cell death, while the resulting inflammatory response and release of DAMPs further amplify the immune component.

The predominance of a particular ROS generation pathway depends on the properties of the PS and the TME, particularly on the oxygen concentration. In Type I reactions, electron transfer occurs between PSs and cellular molecules, resulting in the formation of free radicals, including superoxide anions, hydroxyl radicals, and hydrogen peroxide. Such reactions may proceed under hypoxic conditions. As a result of oxidative damage to proteins, lipids, and nucleic acids, cell death occurs via necrosis (high ROS levels) or apoptosis (moderate ROS levels), accompanied by moderate immune activation [20]. These reactions are particularly suitable for the treatment of poorly oxygenated and deeply located tumors but are associated with a higher risk of off-target toxicity [21,22]. In type II reactions, energy transfer occurs from excited PSs to molecular oxygen, leading to the generation of ^1^O_2_, which exhibits high oxidative capacity [23]. This process is highly oxygen-dependent [20], with apoptosis being the predominant pathway of cell death. A strong immunogenic response is observed. Type II reactions are applied in the treatment of superficially located, well-oxygenated tumors and exhibit limited efficacy under hypoxic conditions [21]. Type II reactions are characterized by more controlled and selective cytotoxicity. Most PSs approved for clinical use generate ROS through type II photochemical reactions, but the type I photochemical reaction may be preferable for therapy of tumors which are hypoxic. This is particularly relevant since the hypoxic TME often acts as a physiological barrier, limiting the oxygen supply necessary for effective singlet oxygen production and leading to suboptimal therapeutic outcomes.

The multifaceted antitumor efficacy of PDT is realized through a complex interplay of three primary, interrelated mechanisms. First, direct cytotoxicity towards malignant cells via ^1^O_2_ generated during the PDT reaction. The induction of different types of cell death depends on the intracellular accumulation of PSs and the intensity of light irradiation: severe photodynamic damage causing disruption of cell membrane integrity triggers necrosis; moderate damage leading to mitochondrial destruction induces apoptosis; and mild damage resulting in lysosomal or endoplasmic reticulum injury promotes autophagy [7]. PDT affects not only tumor cells but is also capable of destroying stromal components of the neoplasm [24]. In addition, effects on the tumor vasculature through ROS-mediated damage to vascular endothelial cells, activation of blood coagulation, platelet aggregation, and thrombus formation, leading to vascular occlusion, persistent hypoxia, and cell death [25]. Finally, stimulation of a systemic antitumor immune response [26] through the initiation of an inflammatory reaction, leukocyte infiltration of the tumor, neutrophil degranulation, and the release of lysosomal enzymes and chemokines, which exacerbate tumor destruction induced by early photodamage. In addition, PDT promotes the repolarization of tumor-associated macrophages from the pro-tumor M2 phenotype to the antitumor M1 phenotype [27,28,29], thereby reprogramming the TME toward an anticancer state.

The ability of PDT to promote macrophage repolarization makes it a key component of combined immunotherapy regimens, enabling modulation of the antitumor immune response.

Overall, PDT exerts a local cytotoxic effect and activates the host immune system, combining direct tumor photo destruction with an immunotherapeutic effect.

### 3.2. Photosensitizers

PSs used in PDT are typically classified into three generations, reflecting the progressive improvement of their photophysical properties, selectivity, and clinical tolerability [30,31].

- First-generation PSs—exemplified by hematoporphyrin derivative (HpD) and Photofrin—are characterized by low chemical purity, peak absorption at a relatively short wavelength of 630 nm, limited tissue penetration depth, a prolonged half-life, and non-specific accumulation in healthy tissues, resulting in cutaneous phototoxicity that may persist for several weeks following treatment. In addition, they exhibit dark cytotoxicity and hydrophobicity, which compromise formulation stability [7,30].
- Second-generation PSs, including chlorins (e.g., chlorin e6 (Ce6), temoporfin), phthalocyanines, and porphyrins such as HPPH, are activated at wavelengths above 650–660 nm and offer improved tissue penetration, higher ^1^O_2_ quantum yield (Φ_Δ_), reduced phototoxicity, better aqueous solubility, and more rapid systemic clearance [30]. Several second-generation agents have advanced to Phase I/II clinical evaluation across multiple tumor types, as detailed in Table 1.
- Third-generation PSs represent chemically modified or nanocarrier-encapsulated second-generation agents, designed to further enhance tumor-selective accumulation. Modification strategies include antibody or peptide conjugation for receptor-targeted delivery, and encapsulation in molecular carriers—liposomes, micelles, quantum dots, dendrimers, polymers, magnetic, gold, and carbon-based nanoparticles—to reduce PS aggregation, prevent premature degradation during systemic circulation, and improve intratumoral bioavailability [31,32]. A distinct subgroup of third-generation systems is specifically engineered to address tumor hypoxia: oxygen-carrying nanosystems (perfluorocarbon-based O_2_ nanocarriers, oxygen-containing nanobubbles, hemoglobin–polymer conjugates) and oxygen-generating nano systems (MnO_2_ nanoparticles, catalase-loaded chitosan–chlorin e6 nanoparticles, biomimetic nano thylakoids) that locally replenish ^3^O_2_ at the tumor site, restoring the conditions required for efficient Type II PDT [33].

Beyond this classical three-generation framework, the current development landscape of PSs is increasingly characterized by three additional design paradigms. First, vascular-targeted photodynamic sensitizers—such as Tookad (WST-11)—leverage the rapid and selective thrombogenic effect on tumor vasculature achieved with short drug-light intervals and near-infrared (NIR) activation, enabling treatment of deep-seated tumors with minimal systemic exposure [17]. Second, activatable (“smart”) PSs are engineered to remain photochemically inert during systemic circulation and to be selectively activated only within the TME in response to specific triggers—pH, redox potential, enzymatic activity, or hypoxia—thereby drastically reducing off-target phototoxicity [34]. Third, two-photon PSs enable precise spatial confinement of photodynamic activation to sub-cellular volumes using near-infrared femtosecond laser pulses, offering unique opportunities for subcellular organelle targeting [3].

Table 1 presents a representative selection of contemporary PSs with limited toxicity profiles and improved therapeutic efficacy, spanning several structural classes and development stages from advanced preclinical to Phase III-validated agents.

**Table 1:** Representative contemporary photosensitizers with limited toxicity and improved efficacy.

PS (Class)	Structural Class	Key Properties	Safety Profile	Current Limitations	Reference
Indocyanine green (ICG)	NIR cyanine dye	Ultra-high selectivity for hepatocellular carcinoma (HCC) cells; dual imaging and PDT utility; activatable at 780–810 nm	Favorable safety profile; adverse event rate extremely low (<0.01%)	Limited NIR light penetration depth (~10 mm); mismatch between standard laser wavelength (823 nm) and ICG tissue absorption peak (805 nm) may reduce in vivo efficacy	[35]
Pheophorbide-a (PPa)	Chlorophyll derivative (chlorin)	Overcomes P-glycoprotein-mediated multidrug resistance; strong absorption at 667 nm; high ^1^O_2_ quantum yield	Low dark cytotoxicity; good biocompatibility; minimal adverse effects compared to synthetic analogs	In vivo validation required; tissue penetration depth may be limiting for bulky or deep-seated tumors	[36]
BSe-B	Selenium-containing dye–biotin conjugate	Two-photon excitation enables precise subcellular spatial control; selective cancer cell targeting via biotin–receptor interaction; differentiates malignant from normal cells	Low dark cytotoxicity; excellent in vivo biocompatibility	Requires specialized femtosecond laser for two-photon excitation; currently at preclinical stage	[37]
Activatable PS A2 (β-carboline/cyanoisoflavone)	Hybrid organic PS	Record-high singlet oxygen quantum yield (Φ_Δ_ = 0.92); effective under hypoxic conditions via combined Type I/II pathways; ferroptosis induction	Minimal toxicity toward normal cells; high tumor selectivity	Long-term safety mechanisms require further study; pharmacokinetics require optimization before clinical translation	[38]
Tookad (WST-11)	Palladium bacteriochlorophyll derivative (VTP agent)	NIR activation at 753 nm; superior tissue penetration; induces rapid tumor vascular thrombosis and ischemic necrosis within minutes of irradiation; Phase III-validated for prostate cancer	5-year follow-up data: acute urinary retention 7.7%; transient hematuria 23%; all events ≤Grade 3; no serious adverse events at 5 years	Evidence is limited to low-risk prostate cancer (ISUP Grade Group 1, PSA < 10 ng/mL, cT1-2a); small study cohorts; ultrasound-guided fiber-optic needle placement required; standardized follow-up protocols lacking	[39]
Zinc phthalocyanine (ZnPc)	Second-generation phthalocyanine	Selective targeting of folate receptor-positive tumors via ligand-functionalized core–shell nanoparticles; large tissue penetration depth due to absorption > 670 nm	Minimizes thermal damage to healthy tissues	Currently at preclinical stage, synthesis of core–shell nanoparticle carriers are technically demanding	[40]
Rose Bengal in exosomes (Er/RB@Exos^Nodal^)	Combinatorial nanoplatform (exosome delivery)	CD47 surface modification enables evasion of mononuclear phagocyte system clearance, increasing intratumoral accumulation; combined chemo-photodynamic and ferroptosis induction	Lower hepatic toxicity compared to non-modified exosome controls	Requires short-wavelength (532 nm) laser; applicable only to superficial tumors or intraoperative settings; exosome formulation manufacturing and standardization are complex	[41]
HPPH (2-[1-hexyloxyethyl]-2-devinyl pyro pheophorbide-a)	Second-generation chlorin	High lipophilicity combined with rapid skin clearance; dual theragnostic utility (fluorescence imaging + PDT); Phase I/II clinical trials completed for esophageal, lung, and head and neck cancers	Minimal and rapidly resolving cutaneous phototoxicity	Limited tumor-cell selectivity; unable to stimulate systemic immune responses; advanced-phase clinical trial data lacking	[42]

Abbreviations: PS—photosensitizer; NIR—near-infrared; HCC—hepatocellular carcinoma; Φ_Δ_—singlet oxygen quantum yield; VTP—vascular-targeted photodynamic; ISUP—International Society of Urological Pathology; PSA—prostate-specific antigen; PDT—photodynamic therapy; ^1^O_2_—singlet oxygen.

The recent developments of PSs follow two primary paradigms: passive and active targeting, both aimed at ensuring selective accumulation within the complex TME. Passive targeting primarily exploits the Enhanced Permeability and Retention (EPR) effect, characterized by the high permeability of abnormal tumor vasculature and the absence of functional lymphatic drainage. This allows nanocarriers, such as liposomes or polymeric nanoparticles (NPs), to sequester within the tumor, improving pharmacokinetic profiles and reducing systemic toxicity [43,44]. While effective for many solid tumors, this approach is limited by the heterogeneity of the EPR effect and the presence of dense extracellular matrices in certain neoplasms, such as pancreatic adenocarcinoma, which hinder NP diffusion [45].

To overcome these barriers, active targeting involves the modification of PSs or nanocarriers with specific ligands (e.g., antibodies, peptides, or small molecules) that bind to receptors overexpressed on tumor cells, facilitating receptor-mediated endocytosis [46]. An illustrative example is the encapsulation of the TPA-DPPy photosensitizer into low-density lipoprotein (LDL) particles to target cells expressing LDL receptors. Such constructs demonstrate high uptake efficiency (~88%) and allow for real-time fluorescence-feedback monitoring [47]

Beyond these approaches, the emergence of ‘Smart’ stimuli-responsive PSs represents a significant shift toward precision PDT. These systems are designed to remain inactive until triggered by specific internal stimuli, such as acidic pH, specific enzymes, or temperature shifts within the tumor site. By minimizing off-target effects and enhancing spatial control, these ‘intelligent’ platforms offer a promising path toward clinically viable and synergistic therapeutic protocols [34].

### 3.3. Limitations of PDT and Strategies to Overcome Them

The hydrophobic nature of PSs leads to their aggregation in aqueous environments, which limits the effectiveness of PDT [48]. In addition, PSs exhibit limited tumor-specific affinity and may damage adjacent healthy tissues during treatment. To enhance selectivity, various smart delivery systems for PSs are being investigated [49]. Surface modification of PSs with various ligands that selectively interact with specific receptors overexpressed on the surface of tumor cells may also promote the accumulation of PSs within tumors [50].

Oxygen is an essential component for triggering the mechanism for PDT activity; however, the active proliferation of malignant cells leads to the formation of a hypoxic microenvironment, which can significantly reduce phototoxic damage mediated by singlet oxygen (^1^O_2_) generation and markedly suppress PDT activity [13]. In turn, PDT itself may further exacerbate tumor hypoxia and result in a decrease in the overall therapeutic effectiveness [14], while hypoxia activates signaling pathways associated with the survival of malignant cells in response to PDT. To overcome this limitation, hypoxia-responsive nanoparticles are being developed for the delivery of oxygen carriers based on perfluorocarbons, as well as nanocarriers loaded with catalase, which locally increase the oxygen concentration within the tumor region [51]. Several studies have demonstrated the potential of using type I PSs, for example, methylene blue, approved for clinical use, for the treatment of hypoxic tumors [52,53]. In addition, catalytic PSs, such as manganese dioxide-modified PSs, are being investigated; these agents are capable of decomposing endogenous hydrogen peroxide to generate oxygen and thereby enhance PDT efficacy in hypoxic areas. The integration of PDT with oxygen-independent therapeutic modalities is also aimed at addressing this challenge. Furthermore, various strategies designed to locally increase tissue oxygenation are being explored, including combination with other forms of energy (X-rays, ultrasound), which may facilitate improved oxygenation or oxygen delivery [54,55].

Blood lipoproteins play a dual role in PDT: they serve as the primary transport system for many PSs while simultaneously imposing key limitations that affect the efficacy and predictability of therapy. The lack of control and variability in delivery of the PSs to tumors directly influence the selectivity of drug accumulation and the final therapeutic outcome. To overcome this limitation, the development of water-soluble PS formulations, targeted PSs delivery systems using nanocarriers, and the administration of agents prior to PDT that transiently increase tumor vascular permeability are being explored, thereby enhancing PS accumulation independently of the lipoprotein-mediated pathway [56].

Additional challenges in the application of PDT include the limited depth of light penetration into tissues, which necessitates invasive light delivery using fiber-optic cables for deep-seated tumors, as well as the need for precise dosimetry. These limitations can be addressed to the application of advanced approaches, including the use of high-energy ionizing radiation sources, the incorporation of nanomaterials responsive to near-infrared light, the development of implantable light-emitting diode (LED) systems, biodegradable fiber-optic devices, and other emerging technologies [57].

Interstitial PDT (iPDT) represents an important advancement for treating large (up to 1 cm thick) or deep-seated tumors that are inaccessible via external light sources. By delivering light directly into the tumor mass through percutaneous needles or anatomical orifices, iPDT minimizes damage to surrounding healthy tissues and preserves the anatomical integrity of organs, which is particularly important in neurosurgery, urology, and head and neck oncology [58,59]. The use of personalized 3D modeling and real-time dosimetry further enhances the precision of this approach [60].

However, iPDT is not without limitations. The primary challenge lies in the complexity of light dosimetry due to tissue heterogeneity and variable PS accumulation. Furthermore, the procedure carries inherent risks, including bleeding, infection, or potential injury to adjacent vital structures, such as blood vessels or organ walls [61].

A critical factor in iPDT is the photothermal effect. Unlike conventional PDT, the high-power density near the fiber tip can lead to significant heat generation. While uncontrolled temperatures exceeding 50–60 °C may result in non-selective thermal necrosis of both tumor and healthy tissues, moderate hyperthermia can be beneficial. Evidence suggests that controlled heating increases cell membrane permeability and boosts the quantum yield of ROS, thereby synergistically enhancing the photodynamic effect [59,62]. To mitigate the risk of excessive heat, modern protocols incorporate real-time temperature monitoring, pulsed irradiation modes, and the use of cylindrical diffusers to ensure uniform light and heat distribution.

While PDT has achieved significant success in dermatology—particularly for treating actinic keratosis, basal cell carcinoma, and other superficial skin lesions—its integration into broader oncology faces several critical hurdles. These limitations can be categorized into four main areas:

1. Fundamental Biological and Physical Constraints: The primary physical barrier is the limited penetration depth of light (typically 5–10 mm), which restricts the treatment of deep-seated solid tumors. Furthermore, the inherent hypoxia of many solid tumors, exacerbated by the oxygen consumption during the PDT process itself, significantly reduces therapeutic efficacy. Insufficient selectivity of conventional PSs remains a concern, leading to risks of skin and ocular phototoxicity, while high lipophilicity often results in poor solubility and bioavailability [63].
2. Technical and Clinical Challenges: Clinical implementation is often hampered by the multi-stage and time-consuming nature of the procedures, requiring significant clinical resources. Patient compliance can be affected by pain during irradiation, which may prevent the delivery of the full therapeutic light dose. Additionally, real-time dosimetry remains complex due to dynamic changes in tissue optical properties during treatment [12,63]. The need for post-treatment pre-cautions (avoiding light exposure) and repeated courses also adds to the clinical burden.
3. Economic and Standardization Barriers: The high cost of specialized PSs, incomplete insurance coverage, and regional disparities in equipment availability limit patient access. Furthermore, the field still lacks universally accepted standardization criteria for treatment protocols [64,65].

Despite these challenges, PDT remains a cornerstone of dermatologic oncology. Its success in this field is driven by the direct accessibility of lesions, the feasibility of local PS application (which eliminates systemic toxicity), and superior cosmetic outcomes compared to invasive surgery. Modern low-intensity irradiation regimens now allow for the painless treatment of large surface areas while stimulating a beneficial immunomodulatory response [66,67].

Overcoming these limitations through advances in PS design, oxygen modulation strategies, and improved delivery systems will be critical for enhancing the clinical efficacy of PDT and expanding its application in oncology.

The impact of tumor hypoxia and the immunosuppressive TME on PDT efficacy extends beyond the monotherapy setting: as detailed in Section 4.2 and Section 5.1.1, respectively, these same factors constitute the primary barriers to synergistic interactions with chemotherapy and immune checkpoint inhibitors, and overcoming them remains the central challenge for all combination strategies reviewed in this manuscript.

### 3.4. Practical “What Not to Do” Principles in PDT

When planning and performing PDT, it is essential to consider a number of limitations that must be observed for patient safety. First and foremost, like any cancer treatment, histological confirmation of the diagnosis is necessary, as the method itself does not provide this possibility, and performing it without a confirmed diagnosis of malignancy may be either excessive or insufficient for the patient. PDT should not be used in cases of allergy or hypersensitivity to the components of the PS [68] or during pregnancy (as safety data for the fetus are lacking). Furthermore, in patients with porphyria, the administration of exogenous PS against a background of high endogenous porphyrin concentrations can trigger a hypertoxic reaction, even with accidental light exposure [69]; therefore, PDT is not recommended for such patients.

In addition to the absolute contraindications mentioned above, relative limitations include [70,71]:

- premature termination of light-protection measures.
- intense physical exertion, or visiting swimming pools and saunas for up to one month;
- sexual contact for up to 4 weeks for patients with cervical cancer.

Since a side effect of PS is increased systemic photosensitivity of the skin and eyes, potentially leading to burns from sunlight or artificial light, patients must follow specific precautions while the PS remains in the body [70]. This includes wearing sunglasses, opaque long-sleeved clothing, wide-brimmed hats, and gloves when outdoors during daylight hours. It is recommended to stay away from windows and avoid tanning beds, dental treatments, surgical procedures, and the use of pulse oximeters.

Regarding diet, it is advisable to consume more vegetables and whole grains to improve bowel function, as well as foods containing beta-carotene, which may reduce photosensitization. Conversely, products that increase light sensitivity—such as figs, celery, spinach, kelp, dragon fruit, and golden snails—must be avoided. These simple clinical recommendations will allow patients receiving PDT to improve their treatment tolerance.

## 4. Combination Strategies of PDT with Anticancer Therapies

### 4.1. Combination of PDT with Anticancer Chemotherapy

**Figure 2:**
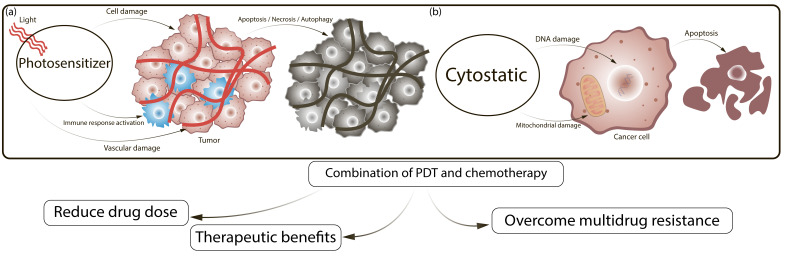
Mechanistic rationale for combining PDT with chemotherapy. (**a**) Principal antitumor mechanisms of PDT: direct tumor cell cytotoxicity via ROS generation, ROS-mediated damage to the tumor vasculature leading to vascular occlusion and ischemia, and induction of a systemic antitumor immune response through immunogenic cell death (ICD). (**b**) Mechanisms of cisplatin-based chemotherapy: DNA crosslink formation leading to replication arrest and apoptosis, modulation of pro-apoptotic signaling pathways, and generation of intracellular oxidative stress. The complementarity of these two mechanistic profiles—spatial selectivity and immune activation of PDT combined with the DNA-damaging and proapoptotic action of platinum-based agents—underpins three key synergistic benefits of their combination: dose reduction in cytotoxic agents without loss of efficacy, reversal of multidrug resistance, and enhancement of overall therapeutic outcomes. Note: PDT—photodynamic therapy; ROS—reactive oxygen species; ICD—immunogenic cell death.

The mechanistic complementarity between PDT and conventional chemotherapy provides a compelling rationale for their combined use, as illustrated in Figure 2. PDT exerts its antitumor effect primarily through localized ROS generation, vascular disruption, and immune activation (Figure 2a), while cisplatin-based chemotherapy acts via a fundamentally different mechanism—covalent deoxyribonucleic acid (DNA) crosslinking, activation of pro-apoptotic signaling, and intracellular oxidative stress induction (Figure 2b). Precisely because these mechanisms are non-overlapping, their combination does not simply add cytotoxic effects but produces genuine synergy: PDT-mediated vascular priming enhances intratumoral accumulation of subsequently administered platinum agents, photodynamic inactivation of efflux pumps and antiapoptotic proteins restores drug sensitivity in resistant cells, and the combination of oxidative and genotoxic damage lowers the threshold for apoptosis induction [7,9]. As a result, synergistic antitumor effects can be achieved at substantially reduced chemotherapy doses, mitigating systemic toxicity while maintaining or enhancing therapeutic efficacy. To exploit these advantages, an increasing number of research groups have combined PDT with chemotherapy across a wide range of tumor models and clinical settings [7,9].

ROS generated during PDT can destroy tumor cells directly by causing apoptosis, necrosis, and/or autophagy. They can also disrupt the tumor vasculature, disrupt the oxygen supply and activate the immune response against tumor cells [72,73] (Figure 2a). The most used chemotherapeutic drugs in cancer treatment today are platinum (II) (Pt (II))-based agents. Their cytotoxic effect is primarily mediate by the formation of intrastrand DNA crosslinks and, to a lesser extent, interstrand crosslinks [74]. Such DNA damage alters the double-helix structure, triggering cell cycle arrest via DNA damage response pathways and ultimately leading to tumor cell apoptosis (Figure 2b). These agents also have known secondary mechanisms of action, such as effects on mitochondrial biogenesis [75] or ROS production [76].

The combination of PDT with chemotherapy has been proposed to enhance the efficacy of low-dose chemotherapy in metastatic forms of breast cancer (BC), lung cancer, cervical cancer, ovarian cancer, head and neck tumors, and cholangiocarcinoma [9,24]. Positive effects of the combined treatment, compared with each modality used alone, have been demonstrated in cell lines, in animal models, and clinical settings [9,77].

For example, exposure to low doses of doxorubicin (DOX) in combination with methylene blue (MB)-mediated PDT in triple-negative BC cell lines (MDA-MB-231 cells) resulted in a significant reduction in cell viability and metabolic activity compared with treatment using the cytostatic agent alone [78]. In studies conducted on HeLa cells (cervical cancer cells), it was shown that the use of low doses of carboplatin in combination with Photofrin-mediated PDT enhanced apoptotic effects compared with carboplatin monotherapy or PDT alone, and also led to increased ROS generation relative to PDT [79]. Furthermore, PDT combined with a low dose of cisplatin may induce apoptosis in HeLa cells by enhancing molecular and functional alterations in the p53 signaling pathway compared with each treatment modality applied individually [9].

According to the study by de Freitas L. M. et al. (2017), the sequence of combined therapy applied to tumor cells yields different outcomes depending on the PS used [80]. Specifically, low doses of cisplatin act as sensitizing agents for Photogem, whereas MB increases the sensitivity of tumor cells to cisplatin [80]. The same authors demonstrated that PDT monotherapy predominantly induces necrotic cell death (two-thirds of dead cells), whereas cisplatin primarily triggers apoptosis. Combined therapy results in a higher proportion of apoptosis-like or necrosis-like cell death across different malignant cell lines compared with monotherapy. This study also confirms a synergistic effect between PDT and cisplatin.

It should be noted that 3D human cell culture models are among the most suitable systems for studying the effects of PDT. These models mimic the complex structure of human tumors, thereby bridging the gap between traditional cell cultures and in vivo models. They replicate tumor architecture and heterogeneity more accurately than conventional 2D cultures [81] while allowing for high-throughput testing of numerous therapeutic combinations compared to in vivo studies [82]. Furthermore, these models are compatible with various diagnostic imaging techniques. Depending on the research objectives, various platforms can be utilized, including non-adherent spheroids, matrix-adherent or matrix-embedded microtumors, and patient-derived cancer organoids obtained from surgically resected tissues [4].

For instance, a study by Broekgaarden et al. (2018) using organotypic 3D cultures modeling metastatic pancreatic cancer demonstrated the superior efficacy of neoadjuvant verteporfin-PDT combined with oxaliplatin compared to either monotherapy [83]. This combination therapy successfully overcame chemoresistance in AsPC-1 organoids derived from human metastatic pancreatic cancer cells; specifically, neoadjuvant PDT increased oxaliplatin efficacy in suppressing cell viability by 25-fold (*p* < 0.0001). The authors suggest that this effect is linked to the direct induction of apoptosis and disruption of cellular redox homeostasis by PDT. Subsequent DNA damage caused by oxaliplatin leads to genomic instability which, combined with the unbalanced redox state, triggers cell death [83].

Additionally, in vivo orthotopic pancreatic cancer models have shown that PDT leads to a downregulation of the ABCG2 multidrug transporter, resulting in increased cytostatic retention and enhanced efficacy [84]. A similar mechanism has been demonstrated for oxaliplatin following PDT in colorectal cancer cells [85]. Thus, the use of such models enables the evaluation of the temporal kinetics of interactions between mechanistically distinct treatment modalities.

However, a limitation of both spheroid and more complex hydrogel models—which reproduce metabolic changes resulting from tumor-stroma interactions [86]—is the lack of fluid flow. The introduction of fluid dynamics via microfluidic platforms [4] may induce resistance to cytostatic agents while simultaneously enhancing the delivery of photo immunoconjugates and the overall efficacy of photoimmunotherapy [87].

Animal studies have also been conducted. In an experimental study by Anand S. et al. (2019), conducted using a triple-negative BC model in female nude mice inoculated with transgenic murine 4T1 cells expressing luciferase, the efficacy of capecitabine in combination with 5-aminolevulinic acid (5-ALA)-mediated PDT was evaluated [88]. The combined therapy was shown to selectively increase protoporphyrin IX levels in 4T1 tumors, enhance tumor cell death, and more effectively inhibit primary tumor growth and metastasis compared with PDT monotherapy [88]. In a study by J.C. Cacaccio et al. (2022), performed in female SCID mice bearing small-cell lung cancer (SCLC) 14541 tumors, a synergistic effect of PDT combined with DOX chemotherapy was demonstrated with respect to achieving the rate and duration of a complete treatment response [89].

The efficacy of chemophototherapy can be significantly influenced by the time interval between cytostatic administration and the PDT procedure. In the study Luo D. et al. (2018) presented a semi-mechanistic pharmacokinetic/pharmacodynamic model for the quantitative analysis of PDT-induced vascular permeabilization and subsequent enhancement of antitumor efficacy [90]. The study utilized mice bearing subcutaneous human pancreatic adenocarcinoma xenografts (MIA PaCa-2) treated with long-circulating LC-Dox-PoP liposomes loaded with doxorubicin (DOX) and incorporating 2 mol% porphyrin-phospholipid in the bilayer. PDT using 2 mg/kg LC-Dox-PoP was shown to increase vascular permeability, resulting in a 12.5-fold increase in doxorubicin influx into the tumor [90]. Notably, shorter drug-light intervals (0.5–3 h) ensured higher intratumoral concentrations of the cytostatic and improved therapeutic outcomes compared to longer intervals.

However, vascular density and diameter can vary significantly between different tumor types. In a study by Ghosh and Lovell (2021), the use of dual laser irradiation in two models of subcutaneous human pancreatic tumors in mice demonstrated superior results [91]. The protocol involved irradiation with a power density of 150 J/cm^2^ one hour after the administration of 3 mg/kg LC-Dox-PoP, followed by a second dose of 50 J/cm^2^ eight hours later, when the highest drug accumulation in the tumor was observed. This dual-treatment approach caused potent tumor ablation and proved more effective than a single laser treatment of 200 J/cm^2^ at either time point [91]. According to Obaid G. et al. (2024), such a strategy allows for the maximum utilization of vascular permeabilization benefits provided by porphyrin-phospholipid liposomes, representing a key aspect of enhancing chemo phototherapy effectiveness [4]. Other factors contributing to improved treatment outcomes include non-ablative modulation of tumor tissue by PDT, reduction in stromal cell density, extracellular matrix remodeling via collagen modulation, and the immunomodulatory effects of PDT [4]. A number of other in vitro and in vivo studies are summarized in Table 2.

Overall, the data in Table 2 demonstrate that the sequence of administration is a key determinant of success, with PDT typically serving as a ‘pharmacokinetic primer’ that increases the intratumoral accumulation of cytostatic drugs.

Despite the promising and encouraging results demonstrated by the combination of PDT and chemotherapy in experimental settings, the use of this combination in clinical practice remains limited. Only a limited number of studies describe the successful application of combined therapy in patients with certain malignant neoplasms. According to the study by Gonzalez-Carmona M.A. et al. (2019), the use of PDT in combination with chemotherapy in 36 patients with unresectable extrahepatic cholangiocarcinoma resulted in significantly longer overall survival compared with chemotherapy alone (*p* = 0.022) [97]. The median survival was 20 months in the combined treatment group (95% CI: 16.38–23.62), 15 months in patients treated with PDT alone (95% CI: 10.02–19.98), and 10 months in the chemotherapy group (95% CI: 8.45–11.55). The PDT plus chemotherapy group also demonstrated higher 1-year (77.7% vs. 34.1%) and 2-year survival rates (27.5% vs. 17.5%) compared with patients receiving cytostatic therapy (gemcitabine and cisplatin). The combined treatment was well tolerated [97]. Undoubtedly, strengths of the study include the significant total sample size of patients with cholangiocarcinoma within a single specialized center (*n* = 353), the favorable survival outcomes in the combination therapy group, the identification of the prognostic significance of PDT in increasing patient life expectancy, and the good tolerability of the treatment. However, the study has several limitations, including the small number of patients enrolled in the chemo phototherapy group and the two control groups (PDT alone, *n* = 34; chemotherapy alone, *n* = 26), as well as its non-randomized retrospective design, which introduces an inevitable selection bias. Furthermore, the long inclusion period (2004–2016) is associated with evolving chemotherapy standards, PDT protocols, and other changes in patient management over the twelve-year span. Finally, the single-center nature of the study suggests that the proposed technique might lead to a higher rate of complications if implemented in a general clinic rather than a highly specialized center.

According to a study by Wentrup R. et al. (2016), PDT combined with chemotherapy in 33 patients with unresectable hilar cholangiocarcinoma was associated with an increase in mean survival to 520 days compared with 374 days in the PDT monotherapy group (*p* = 0.021) [98]. Patients receiving the combined therapy also demonstrated higher 1-year survival rates (88% vs. 58%, *p* = 0.001) and a significantly reduced risk of death (*p* = 0.003), while treatment-related complications occurred with similar frequency in both groups [98]. The strengths of the presented study include a clearly formulated research objective, a significant difference in survival between groups, and an 80% reduction in the risk of death in the combination therapy group compared to the PDT monotherapy group. Furthermore, the study involved patients with a known poor prognosis (Bismuth type III or IV), which enhances the significance of the findings, especially since the groups were well-matched by this criterion. An additional advantage is that the incidence of cholangitis was identical in both groups, indicating that chemotherapy did not increase the procedural risks of PDT. However, similar to previous work, limitations of this study include its non-randomized retrospective design and the heterogeneity of chemotherapy regimens used (gemcitabine as monotherapy or in combination with other cytostatics). The small sample size complicates the interpretation of the results, which, moreover, lack statistical significance. Furthermore, monotherapy is no longer considered the standard of care for this disease. The authors do not provide clear selection criteria for patients assigned to combination therapy versus PDT alone, nor are details of the PDT protocols specified (e.g., PS type, light dose, or frequency of sessions). Given the testing of multiple hypotheses, the issue of multiple comparisons remains, increasing the risk of type I error (false-positive results).

In a retrospective cohort study by Hong M.J. et al. (2014) involving 161 patients with cholangiocarcinoma, 16 patients received combination therapy, while the comparison group consisted of 58 patients treated with PDT alone [99]. The median survival was 538 days (95% CI, 475.3–600.7) in the combination group—consistent with the previous studies—versus 334 days (95% CI, 252.5–415.5) in the PDT group (*p* = 0.05). The one-year survival rate was 93% in the experimental group and 40% in the comparison group; however, two- and three-year survival rates did not differ. The strengths of this work include the larger sample size (*n* = 74) compared to previous studies, a long follow-up period, and more mature data, as all patients in both groups had died by the time of analysis. Multivariable analysis identified combination therapy and receiving more than two PDT sessions as independent predictors of longer survival. The complication rate was low, matching other reports. Nevertheless, this study also shared common limitations: a non-randomized retrospective design, groups that were unbalanced regarding critical prognostic factors (e.g., lymph node metastasis, PDT frequency), and lack of defined criteria for assigning combination therapy. Heterogeneity in cytostatic regimens and the ten-year enrollment period likely reflected changes in standard care protocols. Today, the absence of chemotherapy in the control group is not considered standard. Additionally, the small size of the combination group may have influenced the results. Thus, while this study introduces the dose-effect concept for PDT and shows comparable survival and tolerability to later works, the question of the advantages of PDT combined with modern chemotherapy versus modern chemotherapy protocols alone remains unresolved.

Currently, two clinical trials regarding the use of PDT in combination with chemotherapy for inoperable cholangiocarcinoma have been reported (NCT02082522, NCT04099888) [100,101]. However, the OPUS trial (NCT02082522), a prospective randomized controlled phase III study, was terminated prematurely due to low recruitment and failed to meet its objective [100]. No statistically significant conclusions regarding efficacy could be drawn. The RELEASE trial (NCT04099888), a prospective randomized open-label phase II study, investigated photochemical internalization (PCI) with fimaporfin [101]. Although completed, it did not achieve its goal, failing to show a statistically significant advantage in progression-free survival when adding PCI to standard chemotherapy. Future clinical trials will likely expand our understanding of the roles of PDT and chemotherapy in the management of inoperable cholangiocarcinoma.

In the study by Furukawa K. et al. (2019), PDT in combination with chemotherapy was administered to 12 patients with advanced stage IIIA–IV non-small cell lung cancer (NSCLC) complicated by central airway stenosis [102]. The authors report a reduction in the severity of stenosis to 15% one month after treatment, a median survival of 5.9 months, an overall one-year survival rate of 30.0% [102], and the possibility of avoiding extensive surgery in patients with synchronous tumors. This study combines early and advanced stages, synchronous and metachronous tumors, which complicates the interpretation of the results obtained; in addition, the treatment results for the control group are not presented.

A study by Chhatre S. et al. (2021) included a total of more than 1,048,113 patients with non-small cell lung cancer (NSCLC), among whom 147 patients with stage III–IV disease received combined treatment with PDT, radiotherapy, and chemotherapy, resulting in improved survival compared with other treatment groups [103]. The median survival of patients receiving the combined therapy was 18.6 months, compared with 18.0 months in the radiotherapy plus chemotherapy group, 7.3 months in patients treated with radiotherapy alone, and 16.9 months in patients receiving chemotherapy alone (*p* < 0.0001) [103]. The strengths of this study include its large sample size and representativeness, with data reflecting real-world clinical practice. The addition of PDT to radiotherapy (and chemotherapy) was shown to reduce the risk of death by 50% compared with radiotherapy alone. The use of propensity score matching reduces the risk of selection bias, while the application of validated PDT billing codes based on Medicare/Medicaid data (code J9600 for Photofrin) increases the reliability of identifying patients who received PDT specifically. However, despite the large overall cohort, only 0.03% of included patients received PDT, which raises the risk of residual selection bias; these patients may differ substantially from the remainder even after propensity score adjustment. Key clinical variables are not reported—for example, ECOG performance status, pulmonary function, and precise tumor localization. Core PDT protocol parameters are not described (type of PS, number of treatment sessions, etc.), and no data on quality of life or treatment-related toxicity are provided. Nevertheless, despite these limitations, the study makes a meaningful contribution to the evidence base supporting the efficacy of PDT in combination with radiotherapy and chemotherapy in advanced non-small cell lung cancer (stages III–IV) and identifies a promising treatment option for elderly and comorbid patients.

The study reported in [104], based on a prospective randomized trial enrolling 42 patients, demonstrated that neoadjuvant endobronchial PDT combined with chemotherapy is safe and significantly improves the rate of radical surgical resection in patients with initially unresectable centrally located stage III NSCLC. The objective response rate following neoadjuvant therapy was 90% in the PDT group versus 76% in the control group (*p* = 0.46). R0 resection was achieved in 89% of patients in the combined therapy group compared with only 54% in the control group (without PDT) (*p* = 0.038); R1 resection rates were 11% and 46%, respectively. No serious PDT-related complications were observed. In addition to an optimal study design and well-matched patient groups with respect to age, sex, disease stage, and histological subtype, this study employed a standardized PDT protocol—the procedure was performed prior to each of three chemotherapy cycles. The study also demonstrated a significant increase in the proportion of radical resections, a higher rate of organ-preserving surgical procedures, and the absence of serious PDT-related adverse events. The limitations of the study include the small sample size, its highly specific patient selection criteria, the absence of data on overall survival and progression-free survival, an open-label design that may have influenced surgical decision-making, and its single-center setting, which requires external validation. Overall, this study provides a high level of evidence and, in contrast to previous work, demonstrates the benefits of combined therapy not in the palliative but in the radical treatment setting.

**Figure 3:**
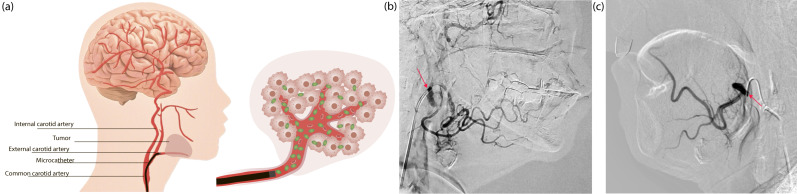
Combined intra-arterial PDT and chemotherapy with image-guided selective microcatheterization of tumor-feeding arteries. (**a**) Schematic overview of the diagnostic and treatment protocol: selective microcatheterization of the tumor-supplying artery under CT fluoroscopy guidance, followed by sequential intra-arterial administration of the PS chlorin e6 (Ce6) and cisplatin at reduced doses, with subsequent transcutaneous laser irradiation of the tumor field. (**b**) Patient 1 (buccal mucosa squamous cell carcinoma)—microcatheter placement at the origin of the facial and lingual arteries branching from the right external carotid artery (ECA). (**c**) Patient 2 (tongue squamous cell carcinoma)—selective microcatheterization of the left facial artery via the left ECA. Red arrows indicate the tip position of the microcatheter at the target vessel. Adapted from [24]. Note: PDT—photodynamic therapy; Ce6—chlorin e6; CT—computed tomography; ECA—external carotid artery.

A clinically important illustration of the PDT–chemotherapy combination is provided by the first-in-human experience reported by Efendiev K. et al. (2025), in which the route of drug administration was specifically optimized to maximize local PS and cisplatin concentrations within the tumor while minimizing systemic exposure [24]. In this study, PDT was combined with selective intra-arterial administration of Ce6 (0.5 mg/kg) and cisplatin at a reduced dose (50 mg/m^2^) in two patients with squamous cell carcinoma of the head and neck—buccal mucosa cancer and tongue cancer, respectively. The intra-arterial approach (Figure 3a), performed under CT fluoroscopy guidance via selective microcatheterization of the tumor-feeding arteries (Figure 3b,c), enabled precise co-delivery of the PS and cytotoxic agent directly to the tumor vascular bed, bypassing systemic dilution and reducing the risk of off-target toxicity.

This strategy exploits the vascular priming mechanism described above: high local Ce6 concentrations in the tumor vasculature, confirmed by spectral and video fluorescence imaging as well as confocal microscopy, ensured rapid and efficient PS accumulation with a shorter exposure time than would be achievable via systemic administration. In both cases, a significant therapeutic effect was achieved, manifested by tumor regression, attainment of grade II–III therapeutic pathomorphosis, epithelialization of the affected areas, and restoration of mucosal integrity. No serious adverse effects or therapy-limiting complications were reported. The authors noted that selective intra-arterial drug administration demonstrated high efficiency of Ce6 accumulation in tumor tissue with a shorter exposure time, which was confirmed by spectral and video fluorescence imaging as well as confocal microscopy. However, the inclusion of only two patients in the study requires further research.

The use of PDT in combination with chemotherapy employing low doses of carboplatin and Photofrin in patients with stage IB1–IB2 cervical cancer demonstrated the effectiveness of this combination in achieving complete tumor regression, long-term disease-free survival, preservation of fertility, and the subsequent ability to successfully carry pregnancies to term and deliver healthy offspring [105]. According to the data reported by the investigators, this approach allows the avoidance of surgical intervention, treatment-related complications, and adjuvant therapy for cervical cancer. However, in addition to the extremely small sample size, there is a lack of data on long-term patient survival and recurrence rates. The procedure protocol is not provided, and there are no toxicity data. Additional examples of the clinical application of PDT combined with cytostatics are presented in Table 3.

Despite these encouraging findings, the clinical evidence bases for PDT–chemotherapy combinations remain methodologically limited. Most published studies are retrospective in design, involve small and heterogeneous patient cohorts, and lack randomized control arms, which substantially constrains the interpretation of survival outcomes and the generalizability of results. Most trials were conducted in patients with unresectable or end-stage disease, making it difficult to isolate the contribution of PDT from that of concurrent systemic therapy. Furthermore, the endpoints reported across studies are inconsistent—ranging from overall survival and objective response rate to pathomorphosis grade—which precludes meaningful cross-study comparisons. The ongoing prospective trials (NCT02082522, NCT04099888, NCT05736406, NCT06381154) are expected to provide higher-quality evidence; however, the absence of standardized PDT dosimetry protocols across institutions remains a key confounding factor even in prospective settings [100,101,107,108].

In 2024, a new clinical trial was registered—NCT05736406 (surgical intervention + intraoperative ALA-PDT (Pentalafen^®^) + temozolomide in patients with newly diagnosed glioblastoma) [107]. This is a non-randomized, open-label, multicenter early-phase (1/2) study with light dose escalation [107]. Its primary objective is to determine the maximum tolerated dose of light for intraoperative ALA-PDT, with the primary endpoint being the presence of dose-limiting toxicity within 28 days post-PDT. Secondary endpoints include progression-free survival at 6 months post-procedure and the overall incidence of adverse events. The main limitations of this study are the small sample size and the absence of a control group; however, a potential future randomized trial based on these results may clarify the efficacy of intraoperative PDT in glioblastoma treatment.

Another study, NCT06381154, is a Phase II protocol aimed at testing the hypothesis that verteporfin-PDT combined with mFOLFIRINOX can enhance the sensitivity of pancreatic cancer to immunotherapy [108]. While this study may provide critical mechanistic data, its results have not yet been published.”

Despite the promising results reported in various laboratory studies, the clinical integration of combined PDT and chemotherapy remains an evolving field that requires large-scale validation. Current evidence suggests that this combined approach is most useful in several specific clinical scenarios:

- Aggressive and Metastatic Neoplasms: In cases where standard chemotherapy alone is insufficient due to rapid progression, PDT can serve as a potent adjunct by modulating the TME. This modulation increases the sensitivity of malignant cells to systemic cytotoxic agents [109].
- Toxicity Limitation in Chemosensitive Tumors: For patients who cannot tolerate full systemic doses of cytostatics, combined therapy allows for a significant dose reduction. The synergistic interaction between PDT and specific drugs ensures therapeutic efficacy is maintained while systemic side effects are minimized [9].
- Overcoming Biological Barriers: PDT has shown particular promise in treating tumors where drug delivery is hampered by physiological barriers. A notable example is glioblastoma, where light-induced disruption of the blood–brain barrier (BBB) facilitates the localized accumulation of chemotherapeutic agents that would otherwise be excluded [110].
- Enhanced Selectivity for Surface and Endoscopic Sites: The use of self-assembling or conjugated PS-drug complexes enables simultaneous action at the tumor site. This is particularly effective for transmucosal delivery in bladder or gastrointestinal cancers, providing high local concentrations with minimal systemic exposure [111].

While the evidence base is still forming, the results of ongoing and future clinical trials are expected to refine the specific patient cohorts and indications for which PDT-chemotherapy combinations will become a primary treatment strategy.

### 4.2. PDT and Tumor Chemoresistance

PDT influences key factors involved in tumor chemoresistance through multiple biological pathways. These include the modulation of transmembrane efflux pumps, the inhibition of antiapoptotic proteins, the elimination of cancer stem cells (CSCs), and the modulation of the TME [112]. While malignant cells can occasionally exhibit adaptive resistance to PDT, the modality generally serves as an effective approach for sensitizing refractory tumors to conventional agents.

A critical mechanism for overcoming resistance involves the reprogramming of mitochondrial energetics. Recent studies suggest that stimulating mitochondrial activity can reverse the metabolic advantages of chemoresistant cells. Methylene blue (MB), a phenothiazine-based PS, has demonstrated the ability to stimulate the mitochondrial electron transport chain and oxidative phosphorylation (OXPHOS) [113]. Preclinical models have shown that MB-mediated metabolic therapy induces apoptosis in cancer cells and suppresses tumor growth in mouse models of ovarian cancer, offering a potential strategy for treating cisplatin-resistant malignancies [114]. Similarly, MB has been shown to suppress glioblastoma proliferation by inhibiting glycolysis and reducing lactate production, effectively shifting tumor metabolism from the Warburg effect toward OXPHOS and increasing overall tumor oxygenation [115,116].

**Figure 4:**
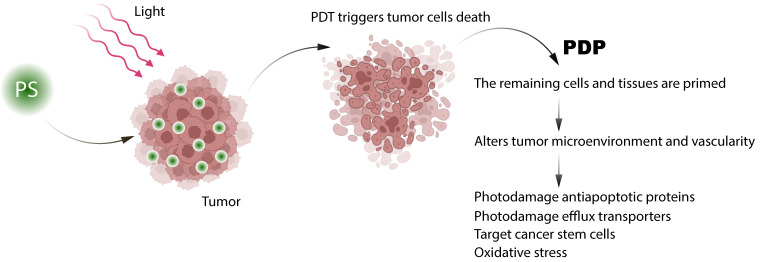
Mechanisms of photodynamic priming (PDP) in the context of combined PDT and chemotherapy. Sub-lethal PDT modulates drug resistance at three complementary levels. At the physical level, ROS-mediated degradation of the extracellular matrix (ECM)—particularly collagen crosslinks—reduces interstitial fluid pressure (IFP) and increases vascular permeability, thereby facilitating the penetration and intratumoral accumulation of subsequently administered chemotherapeutic agents. At the molecular level, photodynamic inactivation of transmembrane efflux pumps (e.g., P-glycoprotein) and pro-survival antiapoptotic proteins (e.g., Bcl-2) restores the sensitivity of resistant tumor cells to cytotoxic drugs. At the immunological level, PDP promotes the infiltration of cytotoxic immune cells and the release of pro-inflammatory cytokines within the TME, contributing to systemic antitumor responses that can target distant disease sites. Together, these mechanisms underlie the rationale for the sequential administration of PDT prior to chemotherapy in combination treatment protocols. Note: PS—photosensitizer; PDT—photodynamic therapy; PDP—photodynamic priming; ECM—extracellular matrix; IFP—interstitial fluid pressure; TME—tumor microenvironment; ROS—reactive oxygen species.

Furthermore, PDT acts as a priming strategy to improve the delivery and efficacy of subsequently administered systemic drugs—a phenomenon collectively referred to as photodynamic priming (PDP). PDP operates through three mechanistically distinct but complementary levels of action, as illustrated in Figure 4. At the physical level, sub-lethal photosensitization induces localized vascular permeability and selectively degrades ECM components—particularly collagen—thereby reducing interstitial fluid pressure (IFP) and creating conditions that favor deep intratumoral drug penetration [117]. At the molecular level, ROS generated during PDP inactivate key mediators of chemoresistance, including transmembrane efflux pumps such as P-glycoprotein and antiapoptotic proteins such as Bcl-2 [112,118], effectively resensitizing refractory tumor cells to cytotoxic agents. At the immunological level, PDP promotes the infiltration of cytotoxic immune cells and the local release of pro-inflammatory cytokines within the TME [26,119], thereby generating systemic effects capable of targeting distant sites of disease. This multi-level reprogramming of the TME by PDP provides the mechanistic rationale for administering PDT prior to chemotherapy in sequential combination protocols [117].

This approach can synergistically attenuate key mechanisms of chemoresistance and improve therapeutic outcomes through combined or non-overlapping action. These properties make this therapy a proper option for inclusion in the arsenal of treatments for various types of cancer. This approach, termed vascular preconditioning, has been shown to increase the accumulation of subsequent therapeutic agents by 3–5-fold, allowing for significantly lower doses of drugs like cisplatin or doxorubicin to achieve comparable efficacy without off-target toxicity [117].

Another important approach is found in PACT, which utilizes prodrugs based on photoactivatable platinum (Pt (II) and Pt (IV)) or ruthenium complexes. Unlike traditional PDT, PACT is often oxygen-independent, making it highly effective for treating hypoxic tumor regions that are traditionally resistant to both radiotherapy and standard PDT [120].

The coordination of these biological effects is increasingly achieved through advanced delivery systems. The implementation of these combinations via nanomedicine is detailed in Section 6.

However, that PDT is not universally resistant-proof, and certain biological contexts can reduce its efficacy or promote adaptive resistance [112]. First, in severely and chronically hypoxic tumors, repeated sub-lethal Type II photosensitization may exert selective pressure favoring the survival of HIF-1α-overexpressing, hypoxia-tolerant cell clones with upregulated pro-survival signaling, thereby reducing the sensitivity of residual cells to subsequent PDT sessions [121,122]. Second, tumor cells exposed to recurrent oxidative stress can upregulate glutathione (GSH) synthesis and other ROS-scavenging systems, progressively attenuating the cytotoxic impact of PDT [123,124]. Third, although PDT initially stimulates antitumor immunity via ICD, this response may be counteracted by immunosuppressive rebound within the TME—including Treg expansion, myeloid-derived suppressor cell recruitment, and upregulation of alternative immune checkpoints—potentially limiting the durability of the antitumor effect and contributing to disease recurrence [26,125]. These considerations underscore the importance of careful patient selection, TME characterization prior to treatment, and the rational sequencing of PDT with complementary agents to preempt or counteract adaptive resistance mechanisms.

A further pharmacological strategy for overcoming PDT resistance involves the combined use of COX-2 inhibitors. Under physiological conditions, COX-2 is expressed at low basal levels in most tissues; however, its expression is rapidly and markedly upregulated in response to inflammatory signals, tissue damage, and cellular stress—conditions that are directly produced by PDT itself. COX-2 catalyzes the synthesis of prostaglandins, including prostaglandin E2 (PGE2), which are key mediators of inflammation, angiogenesis, and cell survival [126]. In the tumor context, chronic COX-2 activation promotes neoangiogenesis, malignant cell proliferation, and suppression of apoptosis. Critically, tumor hypoxia—a hallmark of solid malignancies—can further amplify COX-2 expression via the HIF-1α signaling pathway, creating a self-reinforcing cycle of immunosuppression [127].

PDT itself exacerbates this cycle: ROS-mediated microvascular damage further deepens tumor hypoxia, which in turn drives COX-2 hyperactivation and PGE2 overproduction [128]. Elevated PGE2 recruits regulatory T cells (Tregs) and MDSCs into the TME, establishes immunosuppressive conditions that counteract the antitumor immune response initiated by PDT-induced ICD, and stimulates neoangiogenesis—collectively limiting the durability of the photodynamic effect.

The combination of PDT with COX-2 inhibitors—either selective agents (e.g., celecoxib) or non-selective non-steroidal anti-inflammatory drugs (NSAIDs) (e.g., indomethacin, diclofenac)—addresses these limitations by blocking PGE2 synthesis, resulting in three synergistic effects: (1) reduction in pro-inflammatory cytokines (IL-1β, TNF-α) and suppression of VEGF-mediated neoangiogenesis [129]; (2) reprogramming of the immunosuppressive TME through reduction in Treg and MDSC infiltration and restoration of effector T-cell activity [130]; and (3) enhancement of direct PDT cytotoxicity through activation of apoptotic pathways in tumor cells [129].

At the forefront of this field is the development of hybrid molecules that integrate a PS with a COX-2 inhibitor within a single molecular scaffold. A representative example is the BTDA-IMC nanoplatform, which combines an aggregation-induced emission (AIE) photosensitizer with indomethacin for synergistic enhancement of PDT efficacy [131]. BTDA-IMC provides NIR fluorescence imaging of the tumor and simultaneously suppresses immunosuppressive pathways via COX-2 inhibition, achieving up to 95.71% tumor growth suppression in vivo under laser irradiation with high biocompatibility and minimal systemic toxicity. A complementary approach involves albumin-based nanostructures co-delivering the PS IR780 and diclofenac, which enhance antitumor immune responses, suppress tumor growth, and, when combined with anti-PD-L1 checkpoint blockade, significantly potentiate ICI efficacy [130].

Taken together, COX-2 inhibitors represent a strategically important class of combination partners for PDT, capable of converting PDT from a locally cytotoxic modality into a systemically immunoactivating therapy by dismantling the PGE2-mediated immunosuppressive barrier within the TME.

However, a significant translational gap exists between preclinical success and clinical implementation [132,133]. While murine models often demonstrate near-complete tumor regression, clinical outcomes are frequently less consistent [133]. This discrepancy is largely attributed to the architectural differences between small, superficial animal tumors and bulky, deep-seated human malignancies [134]. In clinical practice, achieving uniform light distribution throughout a large tumor mass remains a primary challenge, as sub-optimal irradiation can lead to regions of inadequate PS activation, subsequently failing to trigger the desired synergistic effect with chemotherapeutic agents [134,135].

Notably, the same hypoxic microenvironment that drives chemoresistance also limits Type II PDT efficacy (Section 3.3) and suppresses the antitumor immune response (Section 5.1.1). This mechanistic triad—the mutual reinforcement of hypoxia, chemoresistance, and immunosuppression—underscores the rationale for multimodal triple combination regimens (PDT + chemotherapy + immunotherapy) in desmoplastic, poorly vascularized tumors such as pancreatic ductal adenocarcinoma, where simultaneous targeting of all three axes may be essential for achieving tangible therapeutic benefit [7,117,133].

## 5. PDT-Driven Immunological Combination Strategies

### 5.1. Combination of PDT with Immunotherapy

**Figure 5:**
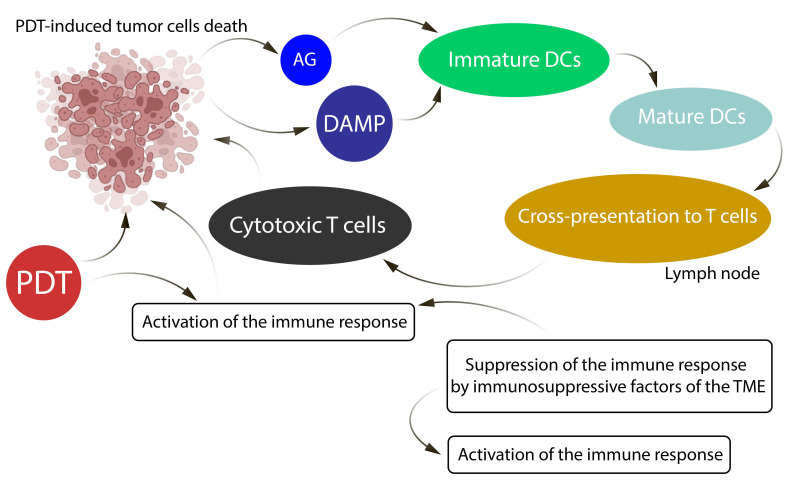
Schematic representation of the immune response following PDT. Induction of ICD by PDT leads to the release of DAMPs and tumor antigens. These are captured by immature DCs, which subsequently mature and migrate to lymphoid organs, including lymph nodes. There, they present (neo)epitopes to naïve T cells. Mature cytotoxic T cells exit the lymph nodes and migrate toward the tumor. Furthermore, activation of a systemic immune response occurs; however, this may be suppressed by immunosuppressive factors within the TME, potentially contributing to disease recurrence or progression. Note: PDT—photodynamic therapy, AG—tumor antigens, DAMP—damage-associated molecular patterns, DCs—dendritic cells, TME—tumor microenvironment.

One of the mechanisms of action of PDT is the induction of a pronounced systemic antitumor immune response, which supports the use of PDT to be a promising option for combination strategies with immunotherapy in the treatment of advanced malignancies. The development of the immune response following PDT is schematically illustrated in Figure 5.

Unlike chemotherapy and radiotherapy, which primarily activate apoptosis at therapeutic doses, PDT is also capable of inducing necrosis and stimulating ICD. A key requirement for ICD is the induction of oxidative stress within the endoplasmic reticulum [136]. ICD can trigger controlled inflammation that recruits immune cells to the TME and promotes their activation. This leads to the effective destruction of the primary tumor and plays a crucial role in forming a long-term specific immune response [5], manifested by the regression of distant lesions never previously exposed to PDT (the abscopal effect) [137]. Factors influencing the probability and potency of the abscopal effect include the immunogenicity of the treated tumor, the dose and type of treatment, as well as the patient’s overall health and immune status [5]. Notably, hypericin is one of the most potent inducers of ICD, even in the absence of irradiation [138]. ICD can be further enhanced by immune checkpoint inhibitors [26] and various PSs, including redaporfin [139].

The stimulation of ICD initiated by PDT is accompanied by the release of tumor-associated antigens (TAAs) and DAMPs. It has been shown that in the PDT-induced immune response, several DAMPs function as critical determinants by increasing the exposure of calreticulin (CRT) and heat shock proteins (HSP70 and HSP90) on the tumor cell surface [140], or by promoting extracellular adenosine triphosphate (ATP) release. CRT can be recognized by phagocytic receptors on DCs, facilitating pro-phagocytic signaling. Antigens released during the ICD process can act as an in situ vaccine, provided they are tumor-specific. Subsequently, TAAs and DAMPs are recognized by DCs, leading to their activation and maturation [141]. Upon reaching the lymph nodes or spleen, DCs present TAAs to naive CD4+ T cells and cytotoxic CD8+ T cells, resulting in their activation and infiltration into the primary tumor and the TME [142]. CD4+ T cells also migrate to and infiltrate metastatic foci, reducing their number [143], while CD8+ T cells increase levels of IFN-γ, which recruits natural killer (NK) cells, memory T cells, and other immune cells, thereby altering the composition of the TME [144].

Furthermore, the HMGB1 protein, released into the extracellular space and interacting with toll-like receptors, stimulates DC maturation and T-lymphocyte formation [145]. ATP released from dying cells stimulates P2RX7 receptors on DCs, which can lead to the formation of the inflammasome complex that directly stimulates the secretion of pro-inflammatory cytokines [146]. PDT also triggers the activation of the complement system and macrophages, promoting increased levels of acute-phase proteins, mannose-binding lectins, and serum amyloid P, which are involved in the recruitment and activation of neutrophils, thereby strengthening the immune response within the TME [119]. Additionally, the induction of ICAM-1 protein expression and MHC-1 presentation during ICD [147] enhances T-cell infiltration into the TME and induces the synthesis of chemokines, such as CXCL10 and CXCL9 [148], which further recruits immune cells to the tumor site.

However, this immune response is not always sufficient to suppress the growth of residual malignant cells following PDT. Moreover, the generated antitumor immunity may be counteracted by various immunosuppressive factors within the TME, thereby contributing to disease recurrence. Specifically, in response to increased levels of interferon-gamma and several other pro-inflammatory molecules (such as interferon-alpha, tumor necrosis factor, and vascular endothelial growth factor), certain cytokines (e.g., IL-4, IL-10) can upregulate PD-L1 expression by the tumor. This promotes immunosuppression and enables the tumor to escape the immune response.

#### 5.1.1. PDT Combined with Immune Checkpoint Inhibitors

In recent years, several aspects of the interaction between PDT and immune checkpoints and their ligands have been elucidated. Ce6-based PDT has been shown to block the PD-1/PD-L1 axis and enhance CD8+ T cell activity without inducing an antitumor immune response [10,149]. MB has also been shown to inhibit PD-1 by blocking the interaction of Y248-phosphorylated immunoreceptor tyrosine-based switch motif of human PD-1 and SHP2; enhance cytotoxic T lymphocyte (CTL) cytotoxicity, proliferation, cytokines; and promotes tumor regression in vivo in mice models [150].

Redaporfin-based PDT can increase PD-L1 expression on tumor cells, whereas ALA-PDT can upregulate PD-1 expression on lymphocytes [151]. In addition, PDT has been demonstrated to increase cytotoxic T-lymphocyte-associated protein 4 (CTLA-4) expression and induce CD80 overexpression in certain tumors, thereby enhancing their immunogenicity. These changes may enhance the efficacy of immune checkpoint inhibitor therapy; however, no correlation between PDT efficacy and PD-L1 expression levels has been observed [152].

The combination of PDT with immune checkpoint inhibitors may overcome the limitations of each modality when used alone and potentiate the antitumor immune response. For example, M.J. O’Shaughnessy et al. (2017) evaluated the efficacy of combining WST11-mediated PDT with immune checkpoint inhibitor therapy (anti-PD-1/PD-L1 antibodies) compared with each monotherapy in a murine (BALB/cAnNCr) orthotopic model of RENCA renal cell carcinoma with pulmonary metastases [153]. The combined treatment resulted in regression of primary tumors, a reduction in the number of pulmonary metastases, and increased animal survival. These effects were absent in the comparator groups. According to the authors, the therapeutic effect was associated with an increased CD8+/regulatory T-cell (Treg) ratio and CD4 + FoxP3−/Treg ratio within primary kidney tumors. In addition, enhanced T cell infiltration of metastatic lesions and reduced Treg proliferation within these foci were observed. Since RENCA cells exhibit low baseline PD-L1 expression, the authors suggest that PDT-induced upregulation of intratumoral PD-L1 expression may serve as a marker of immune system activation, thereby rendering the PD-1/PD-L1 signaling pathway relevant and increasing the likelihood of response to immune checkpoint inhibitor therapy [153].

Alvim R.G. et al. (2021) evaluated the potential of combining PDT with immunotherapy employing a PD-1 inhibitor and an OX40 agonist in a murine (C57BL/6J) model of urothelial carcinoma bearing MB-49 cell allografts [154]. The combined therapy suppressed tumor growth and prolonged survival to a greater extent than PDT or either immunotherapeutic agent used alone. These effects were attributed to enhanced tumor infiltration by CD4+ and CD8+ T cells, activation of cytotoxic T cells and dendritic cells, depletion of the Treg population, a reduction in tumor-associated macrophages, and suppression of myeloid-derived suppressor cells, collectively indicating a strengthened antitumor immune response.

Hao Y. et al. (2022) investigated a combination therapy comprising PDT and immune checkpoint inhibitor–based immunotherapy using a 25% thermosensitive polymeric hydrogel (hydrogel 407) loaded with ICG as a delivery platform in murine (BALB/c and C57BL/6J) models of colorectal cancer (CT26 and MC38 cell lines) [155]. Repeated administration of the combination effectively inhibited colorectal tumor growth and improved survival in tumor-bearing mice compared with immune checkpoint inhibitor or PDT monotherapy [155]. No significant body weight loss was observed during treatment, indicating favorable tolerability. The antitumor effect was further enhanced when antibodies targeting PD-L1 and CTLA-4 were combined. The authors suggest that the observed survival benefit resulted from induction of a robust antitumor immune response and attenuation of immunosuppression within the TME [155]. Additional experimental studies investigating combinations of immune checkpoint inhibitors and PDT are summarized in Table 4.

Across the experimental studies summarized in Table 4, a consistent finding is that the combination of PDT with anti-PD-1/PD-L1 agents produces abscopal effects—suppression of non-irradiated distant tumors—in bilateral implantation models across multiple tumor types (colon, breast, pancreatic, urothelial) [153,154,155,156,157,158,159,160]. This convergence strongly supports the hypothesis that PDT-induced ICD is mechanistically sufficient to prime systemic antitumor immunity when combined with checkpoint blockade. However, a diverging and underappreciated finding concerns the timing and magnitude of PD-L1 upregulation after PDT: while some studies report transient PDT-induced PD-L1 upregulation that facilitates subsequent ICI efficacy [153], others find that PDT-induced hypoxia via HIF-1α can sustain prolonged PD-L1 expression through independent signaling pathways [133], potentially attenuating the T-cell response rather than amplifying it. This divergence has direct clinical implications: it suggests that the optimal inter-treatment interval between PDT and ICI administration may be tumor-type and PS-specific, and requires prospective validation rather than empirical selection.

The potential use of PDT in combination with immunochemotherapy is also being investigated in clinical oncology. Santos L.L. et al. (2018) reported regression of locally advanced squamous cell carcinoma of the floor of the mouth after treatment with redaporfin-mediated PDT combined with nivolumab in a patient who had progressed following radiotherapy, targeted therapy, chemotherapy, and surgical intervention [161]. In addition, a clinical trial was registered at Lanzhou University by Yu Y. et al. (2023) to evaluate the potential for improving treatment outcomes in patients with advanced or metastatic gastric cancer and gastroesophageal junction cancer using PDT in combination with immunochemotherapy [162]. The results of this study have not yet been reported in the available literature. Additional clinical studies investigating the use of PDT in combination with immune checkpoint inhibitors are presented in Table 5.

The combination of immuno-oncology agents with PDT represents a promising antitumor therapeutic strategy, enhancing the efficacy of PDT and expanding its clinical applicability.

The immunomodulatory effects of PDT described in this section are inextricably linked to tumor oxygenation: in severely hypoxic tumors, PDT-induced ICD is attenuated due to reduced ROS generation, while HIF-1α-driven PD-L1 upregulation may sustain T-cell exhaustion, potentially limiting the efficacy of checkpoint blockade [133]. Conversely, ICI-mediated T-cell activation can itself normalize the TME by promoting vascular remodeling and improving tumor oxygenation—creating a positive feedback loop that further enhances PDT efficacy in subsequent treatment cycles [4].

#### 5.1.2. Combined PDT and Targeted Therapy: Photoimmunoconjugates

To enhance the selectivity of PSs, their conjugation with monoclonal antibodies or other immunomodulatory compounds exhibiting high affinity for the TME has been explored, leading to the development of photoimmunoconjugates. These conjugates enhance the efficacy, specificity, and cytotoxicity of PDT, while overcoming delivery barriers associated with the hydrophobic nature of PSs [5]. Various technical strategies are available for creating these conjugates, including both chemical synthesis and genetic engineering approaches.

For example, conjugation of a PS with targeting peptides has demonstrated enhanced selectivity and therapeutic efficacy in triple-negative breast cancer cell lines (MDA-MB-453 and MDA-MB-231) [165]. Cellular uptake of the conjugates correlated with epidermal growth factor receptor (EGFR) expression levels. PDT induced apoptotic cell death in MDA-MB-453 cells, whereas necrotic changes predominated in the MDA-MB-231 cell line. The conjugates significantly inhibited migration of MDA-MB-231 cells with high EGFR expression, indicating a reduced metastatic potential. The authors highlight the potential of EGFR-targeted porphyrin–peptide conjugates as promising PDT agents for the treatment of triple-negative breast cancer.

Otvagin V.F. et al. (2024) investigated the synthesis of a conjugate based on a chlorin-e6 photosensitizer and a derivative of the tyrosine kinase inhibitor cabozantinib, linked by a β-glucuronidase-responsive linker [166]. The study demonstrated that an enzymatically cleavable conjugate effectively inhibited the proliferation of malignant cells (human A-431 tumor cell line) at submicromolar concentrations in vitro, and also suppressed tumor growth through the activation of both photodynamic and targeted therapy in 3D models [166].

Increasing attention has focused on antibody conjugates of IR700 (IRdye700DX), a near-infrared PS that enables highly specific, ligand-mediated delivery of the PS. Upon irradiation, these conjugates selectively induce rapid necrotic cell death in antigen-bearing tumor cells, such as those expressing EGFR or HER2. It is proposed that infrared radiation induces photochemical cleavage of hydrophilic axial ligands from the silicon phthalocyanine core of the IR700 molecule. This reaction triggers a rapid transition of the conjugate from a hydrophilic to a hydrophobic state, leading to its deposition and aggregation on the cell surface, disruption of cell membrane integrity, water influx, and immediate necrosis. This mechanism of membrane permeabilization remains effective even under hypoxic conditions, distinguishing IR700 conjugates from conventional Type II PSs [167,168]. A representative example is the trastuzumab–IR700 conjugate, in which infrared irradiation induces a photochemical switch of the compound from a hydrophilic to a hydrophobic state, promoting aggregation on the cell surface and direct membrane disruption [169].

Monoclonal antibody-based conjugates may face certain limitations [5]. Due to their relatively large molecular size, these compounds exhibit limited penetration into dense, deep-seated, and poorly vascularized tumors. They also tend to persist in the serum and are cleared slowly, which delays the optimal start time for PDT and complicates optical imaging. Furthermore, slow clearance increases the likelihood of side effects and leads to non-specific uptake by the liver and the reticuloendothelial system. Smaller molecules may overcome these limitations [170]. For instance, antibody fragments such as scFv (single-chain Fv fragments) possess a greater and faster capacity for tissue penetration, more rapid blood clearance, and significantly lower renal uptake [171]. PS2:scFv conjugates, synthesized using 5-(4-isothiocyanatophenyl)-10,15,20-tris-(4-N-methylpyridiniumyl) porphyrin trichloride, have demonstrated selective cytotoxic activity in colorectal cancer cell lines (Caco-2) by inducing apoptosis.

The potential for using immunoconjugates in clinical practice is actively being explored. For example, the multicenter, randomized, open-label Phase 3 ECLIPSE study (NCT06699212), which commenced in 2024 [172], is designed to evaluate the efficacy of the cetuximab-IR700 photosensitizer conjugate (ASP-1929) in combination with anti-PD-1 therapy (pembrolizumab) in patients with recurrent head and neck squamous cell carcinomas (HNSCC). The recruitment of 412 anti-PD-1/PD-L1-naive patients with CPS ≥ 1 is planned across 22 research centers. The study aims to determine whether the combination of ASP-1929 PIT and pembrolizumab is superior to the control group (Standard of Care, SOC) in terms of overall survival. This study may be pivotal for regulatory approval as results will be compared against the first-line standard (pembrolizumab monotherapy for CPS ≥ 1 or combination with chemotherapy in a 1:1 ratio). This trial may support a new treatment standard for locally recurrent head and neck cancer, potentially making ASP-1929 PIT the first approved photoimmunotherapy in oncology. A planned interim analysis will allow for the termination of one of the two experimental groups to select the optimal dose for continued therapy. While the study features a clearly defined target population, logical design, and validated quality-of-life questionnaires, its open-label design introduces a risk of selection bias in treatment termination and symptom assessment. Additional limitations include the heterogeneity of the control group regarding immuno- and chemotherapy regimens, the lack of mandatory EGFR expression verification in the inclusion criteria, a long 7-day interval between ICI therapy and PDT, and the absence of mandatory post-PDT biopsies. Results from this study have not yet been published.

Another single-arm Phase 1b/2 dose-escalation clinical trial, NCT03052127, evaluated the recombinant conjugate AU-011 in combination with PDT in patients with small primary choroidal melanoma [173]. AU-011 is a conjugate consisting of a virus-like particle (VLP) and the phthalocyanine IRDye 700DX. It was previously shown [174] that AU-011 possesses potent and selective anticancer activity both in vitro and in vivo, with the binding specificity of the viral particles mediated by heparan sulfate proteoglycans on the surface of tumor cells. The primary advantage of the NCT03052127 study is its potential for an organ- and vision-preserving approach, whereas the current standards remain brachytherapy or enucleation. The study defined clear endpoints for safety and efficacy and represents one of the first human clinical trials of VLP-photosensitizer technology. However, the small sample size, lack of a control group, and short follow-up period are insufficient to draw definitive conclusions regarding efficacy. Although the study is complete, the findings have not yet been published.

Thus, the use of immunoconjugates, as well as the combination of photodynamic therapy with immune checkpoint inhibitors, remains an active area of experimental and clinical research. The integration of such drugs into routine clinical practice remains a future clinical prospect.

### 5.2. PDT in the Development of Antitumor Vaccines

The induction of ICD by PDT suggests that tumor cells treated with this modality can be used to develop of antitumor vaccines. DC-based vaccines appear particularly promising. For example, in one study [175], administration of a DC-based vaccine generated by co-culturing dendritic cells with a murine GL261 glioma cell line previously subjected to hypericin-mediated PDT conferred protective immunity against orthotopic implantation of parental cells in syngeneic mice. This effect was attributed to a reduction in Tregs, ROS generation during ICD of glioma cells, increased expression of DAMPs, and enhanced tumor infiltration by CD8+, CD4+, and Th17 T cells. The resulting DC vaccine acted synergistically with temozolomide chemotherapy, extending survival in mice with high-grade gliomas by approximately 300% and achieving long-term survival in about 50% of animals.

According to Vedunova M. et al. (2022), DC-based vaccines loaded with PDT-treated glioma cells promotes the induction of Th17-mediated immunity and may represent a promising therapeutic strategy for glioma [176].

Trempolec N. et al. (2022) employed PDT to induce ICD in mesothelioma cells followed by the use of tumor cells lysates for DC priming [177]. Administration of this DC-based vaccine to mice (BALB/CByJ strain) with peritoneal mesothelioma resulted in significantly prolonged survival compared with the control group receiving anti-CTLA-4 antibodies. The authors attribute this therapeutic effect to enhanced proliferation and increased cytotoxic activity of CD8+ and CD4+ T cells, as well as elevated infiltration of mesothelioma by interferon-gamma (IFN-γ)-expressing T cells. In addition, upregulation of CCR7 receptors on dendritic cells was observed, accompanied by rapid DC chemotaxis toward lymphatic vessels and tumor-draining lymph nodes.

Recent studies have significantly expanded the therapeutic potential of PDT-derived vaccines, including synergy with immune checkpoint inhibitors and the development of in situ vaccination platforms. Li et al. (2022) developed a photodynamically sensitized dendritic cell (PDT-DC) vaccine in a murine model of HNSCC [178]. The vaccine markedly enhanced the antitumor efficacy of anti-PD-L1 monoclonal antibody therapy, resulting in enhanced T-cell activation, tumor regression, and the induction of systemic immunity [178].

Furthermore, innovative in situ approaches eliminate the need for ex vivo cell processing steps. Liu et al. (2022) demonstrated that endoplasmic reticulum (ER)-targeting PDT using Par-ICG-Lipo nanoparticles induces robust ICD directly within the tumor tissue [179]. This results in the release of DAMPs and tumor antigens in vivo, effectively converting the tumor itself into a therapeutic in situ vaccine. When combined with dendritic cells, the strategy elicited potent systemic antitumor immune responses and offers a highly promising platform for clinical translation for personalized cancer vaccination [179].

Yang et al. (2024) proposed the use of PDT for the development of an antitumor vaccine derived from tumor antigens extracted from malignant tissues obtained following cytoreductive surgery [180]. In this approach, the authors incubated mucoadhesive nanoparticles containing a low-molecular-weight PS with a suspension of tumor cells, followed by irradiation with a 405 nm laser to induce ICD [180]. Vaccine administration was not associated with significant toxicity and elicited a robust tumor-specific immune response, resulting in effective suppression of postoperative tumor recurrence and metastasis. The authors suggest that such a vaccine offers opportunities for personalized therapeutic applications and may be used not only as a standalone treatment but also in combination with cytostatic agents and immune checkpoint inhibitors.

Despite the promising data from DC-based vaccines, several limitations remain [181]. The magnitude of the immune response is highly dependent on the patient’s baseline immune status, which is often severely compromised in late-stage oncology patients—the primary cohort in most clinical trials [182]. Furthermore, the lack of standardized protocols for ‘antigen harvesting’ and the optimal timing between PDT-induced ICD and vaccine administration introduces variability in outcomes, making it difficult to compare efficacy across different clinical studies [183,184].

## 6. Nanotechnology as a Unified Platform for Synergistic PDT Combinations

Nanotechnology serves as a key technological bridge that enables the co-delivery and synchronized action of PSs and pharmacological agents. By integrating diverse therapeutic cargos into a single nanoplatform, it is possible to optimize pharmacokinetics, exploit the Enhanced Permeability and Retention (EPR) effect, and implement “smart” responsive behaviors.

### 6.1. Smart Nanoplatforms for Co-Delivery

**Figure 6:**
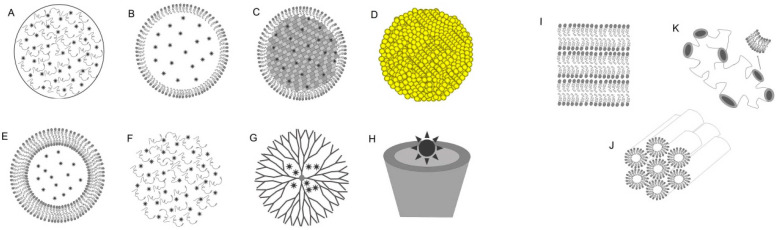
Structural diversity of nanotechnology-based delivery systems for PDT combination therapy. The principal nanocarrier architectures currently employed for PS co-delivery include: (**A**) polymeric nanoparticles (PNPs)—versatile platforms for stimuli-responsive drug release; (**B**) nanostructured lipid carriers (NLCs) and (**C**) solid lipid nanoparticles (SLNs)—lipid-based systems offering high PS encapsulation efficiency and controlled release; (**D**) gold nanoparticles (AuNPs)—inorganic platforms with tunable plasmonic properties enabling photothermal synergy; (**E**) liposomes—the most clinically advanced nanocarrier class, with established safety profiles and scalable manufacturing; (**F**) hydrogels—injectable depot systems enabling sustained local PS release; (**G**) dendrimers—branched macromolecules with precise molecular weight and high surface functionalization capacity; (**H**) cyclodextrins—inclusion complexes that improve PS aqueous solubility and bioavailability; and (**I**–**K**) liquid crystalline mesophases (lamellar, hexagonal, and cubic)—self-assembled lipid matrices with sustained-release properties. Among these, liposomal systems (**E**) are the most extensively studied in PDT–drug combination protocols, as detailed in Table 6. Adapted from [185]. Note: PS—photosensitizer; PNPs—polymeric nanoparticles; NLCs—nanostructured lipid carriers; SLNs—solid lipid nanoparticles; AuNPs—gold nanoparticles.

The development of multifunctional nanocarriers has substantially advanced the design of combined PDT–drug protocols by enabling the synchronized co-delivery of PSs and pharmacological agents within a single platform. These “smart” systems are engineered to maintain fixed PS-to-drug molar ratios during systemic circulation—preventing premature dissociation and differential clearance—and to provide stimulus-triggered release specifically upon reaching the TME or following external light irradiation. Triggering stimuli exploited in current platforms include the acidic tumor (pH-responsive polymers), elevated intracellular glutathione concentrations (disulfide-bond cleavage), overexpressed tumor-associated enzymes (e.g., β-glucuronidase-responsive linkers), and direct photocleavage upon PS activation (Section 6.3). The structural diversity of nanocarrier architectures currently employed for PS delivery is illustrated in Figure 6, ranging from lipid-based systems with established clinical precedents to stimuli-responsive polymeric constructs and inorganic platforms with tunable optical properties [185].

Figure 6 illustrates the structural diversity of nanocarrier platforms available for PS delivery. Among these, liposomal systems (E) are the most clinically advanced due to their established safety profile and scalable manufacturing, whereas polymeric nanoparticles (A) and dendrimers (G) offer greater versatility for stimuli-responsive drug release, as discussed in Section 6.3.

A representative example is the Combo-NP platform, composed of a biodegradable NIR-II polymer and the tyrosine kinase inhibitor Lenvatinib [186]. Beyond its role as a carrier, the nanostructure itself is engineered with disulfide bonds for responsive degradation. Such platforms facilitate several synergistic effects:

- Vascular Normalization: Agents like Lenvatinib delivered via nanoparticles alleviate tumor hypoxia, thereby providing the oxygen necessary for enhanced PDT efficacy.
- Immune Activation: Co-delivery systems can trigger ICD, promote dendritic cell maturation and increase the infiltration of cytotoxic T lymphocytes.
- Reduced Toxicity: As seen with PDA-coated liposomes delivering doxorubicin and ICG, nanocarriers significantly reduce off-target effects, such as cardiotoxicity, while achieving complete tumor regression [187].

The versatility and clinical potential of these lipid-based and polymeric systems are further supported by numerous experimental studies. A comprehensive summary of representative nanoplatforms for co-delivery, including their specific components and therapeutic outcomes, is provided in Table 6.

**Table 6:** Studies involving liposomal systems.

Photosensitizer	Cytostatic	Tumor/Effect	Reference
Pyrolipid, pNALs	CA4P	MCF-7 breast cancer; BALB/c nude mice. Preferential accumulation in tumors; inhibition of tumor growth after PDT with reduced laser irradiation intensity and lower cytostatic dose	[188]
ICG-Lipo-PTX	Paclitaxel	KPL-1 breast cancer; BALB/c mice. Suppression of tumor growth; increased tumor necrosis area in the combined therapy group; inhibition of contralateral tumor growth; immunomodulatory effect characterized by increased interferon-γ and interleukin-2 secretion with suppressed interleukin-10	[189]
Verteporfin, PEGylated nanoliposomes	Oxaliplatin	Orthotopic PANC-1 pancreatic cancer model; mice. Tumor growth suppression in 58% of observations in the combined therapy group; increased intracellular retention of oxaliplatin after PDT	[190]
ICG-Lipo-C&D	Carboplatin with Docetaxel	Colon cancer (Colon-26 model); CDF1 mice. Enhanced antitumor effect in the combined therapy group; increased expression of immune-related genes and decreased expression of cytoskeleton-associated genes	[191]
GA/RGD-DOX/ICG-Lips	Doxorubicin	Hepatocellular carcinoma; mice. Overcoming drug resistance and reduced systemic toxicity; increased intracellular concentration of DOX; dual targeting strategy	[192]

Abbreviations: pNALs—photoactivable nanoliposomes; ICG-Lipo-PTX—indocyanine green (ICG)-modified liposomes in which paclitaxel (PTX); ICG-Lipo-C&D—liposomally formulated indocyanine green derivative (ICG-Lipo) that encapsulated carboplatin and docetaxel (C&D); GA/RGD-DOX/ICG-Lips—glycyrrhetinic acid (GA)/tripeptide sequence arginine-glycine-aspartate (RGD)-targeted and doxorubicin (DOX)/indocyanine green (ICG)-loaded liposomes (Lips); PDT—photodynamic therapy.

The translational feasibility of the advanced nanoplatforms listed in Table 6 faces significant hurdles beyond therapeutic efficacy [193]. Most multifunctional nanocarriers suffer from high synthetic complexity, which complicates large-scale, GMP-compliant (Good Manufacturing Practice) production and limits batch-to-batch reproducibility [194,195]. Additionally, the long-term safety profiles of non-biodegradable components and the potential for off-target accumulation in the liver or spleen remain critical concerns for regulatory approval [196]. Transitioning from ‘bench to bedside’ will require a shift toward simpler, more robust designs that prioritize scalability and predictable pharmacokinetics over multi-component complexity [193].

### 6.2. Nanotechnology as a Unified Platform for Synergistic PDT Combinations

The clinical efficacy of the EPR effect is often inconsistent due to the physiological barriers within the TME. High IFP and a dense, desmoplastic stroma—characterized by cross-linked collagen fibers and an irregular ECM—create a physical shield that prevents the deep penetration and uniform distribution of nanoparticles into the tumor core [112]. In addition, tumor vasculature is highly heterogeneous, with irregular vascular spaces distributed unevenly throughout the tumor mass [7].

Recent strategies employ sub-therapeutic PDT as a potent “pre-conditioning” or “vascular priming” tool to modulate these barriers. Low-dose photosensitization induces transient gaps between endothelial cells and selectively degrades tumor ECM components, specifically collagen and hyaluronan. For instance, preliminary irradiation of subcutaneous prostate adenocarcinoma in mice has been shown to increase the accumulation of subsequent nanomedicines, such as Doxil, by 3–5-fold [117]. This effect was accompanied by a twofold reduction in tumor collagen content and a significant decrease in ECM density in the subendothelial region, effectively lowering the IFP.

By reducing these physical barriers, PDT-mediated preconditioning allows for a significant reduction in the systemic dose of toxic chemotherapeutic agents while maintaining or even enhancing therapeutic outcomes. This approach is particularly promising for treating desmoplastic tumors, such as pancreatic or breast cancer, where traditional drug delivery often fails due to poor stromal penetration [117].

### 6.3. Targeted and Stimuli-Responsive Systems

To achieve precise spatiotemporal control, advanced nanoconstructs are increasingly engineered with targeting moieties and stimuli-responsive elements. These “smart” features ensure that the therapeutic payload is released only under specific conditions, minimizing off-target effects on healthy tissues.

Targeted Delivery: Nanoparticles functionalized with ligands such as monoclonal antibodies (e.g., against EGFR or HER2), folic acid, or specific peptides allow for the active recognition of tumor-specific biomarkers [6,165]. This ligand-mediated endocytosis increases the intracellular concentration of both the PS and the drug cargo. For example, EGFR-targeted porphyrin–peptide conjugates have shown a direct correlation between receptor expression and cellular uptake, significantly enhancing cytotoxicity and inhibiting migration in triple-negative breast cancer models [165].

Stimuli-Responsive Release: The next generation of nanomedicine utilizes the unique biochemical profile of the TME or external triggers to initiate drug release:

- Enzymatic Triggers: Linkers sensitive to overexpressed tumor enzymes, such as β-glucuronidase, allow for the selective activation of conjugates within the malignant tissue. A notable example is the conjugate of chlorin-e6, and a cabozantinib derivative linked by a β-glucuronidase-responsive moiety, which effectively inhibits tumor growth in 3D models [166].
- Light-Triggered Release: Photo-cleavable linkers or light-sensitive polymers enable the precise release of encapsulated drugs only upon irradiation at a specific wavelength. This approach is further exemplified by PACT, where light triggers the activation of oxygen-independent prodrugs, such as platinum (IV) complexes, directly at the tumor site [120].
- Redox and pH Sensitivity: Exploiting the high GSH concentration or the acidic pH characteristic of the tumor interstitium ensures that the nanocarrier disassembles only after reaching the target cell, preventing premature drug leakage during circulation [186].

The integration of these functionalities into a single, cohesive regimen represents the future of personalized oncology, where nanotechnology serves as the platform to unify PDT with chemotherapy and immunotherapy into a synergistic, highly selective treatment modality.

## 7. Future Perspectives and Research Directions

Encouraging preclinical data suggest that, with optimized irradiation protocols and refined antitumor drug dosing regimens, may enable enhanced therapeutic efficacy while enabling dose de-escalation of both cytostatic agents and PSs. Such improvements may reduce treatment-related toxicity, thereby improving patients’ quality of life and potentially extending survival. Moreover, robust therapeutic responses may facilitate down-staging of previously inoperable tumors, allowing patients access to more effective curative or consolidative treatments.

Although the number of clinical studies combining PDT with cytostatic agents remains limited, the available evidence indicates that these favorable outcomes are clinically achievable. Randomized controlled trials will be particularly valuable in establishing the role of PDT-based combinations in tumors that respond poorly to standard therapies. Future studies should also focus on clarifying the efficacy and safety of PDT when paired with various immunotherapeutic agents in solid tumors. In addition, deeper investigation is needed into the specific immunomodulatory effects of PDT in cancer patients.

The gap between preclinical and clinical outcomes is modality-specific and instructive. For PDT + chemotherapy, preclinical models consistently demonstrate near-complete tumor regression with 3–5-fold increases in drug accumulation [117]; however, the largest clinical dataset showed only a modest 1.7-month survival advantage for the combination arm [103]. This discrepancy is largely attributable to the fact that murine subcutaneous tumors—the dominant preclinical model—lack the fibrous, poorly vascularized stroma that characterizes human cholangiocarcinoma or NSCLC, where the EPR effect and vascular priming are far less predictable [133]. For PDT + immunotherapy, preclinical models employing bilateral tumor implantation (e.g., CT26, 4T1) impressively demonstrate abscopal regression of non-irradiated lesions [157,158]; however, clinical evidence in humans remains limited to case reports [164]. The immunological conditions that enable abscopal effects in murine models, high tumor mutational burden and a responsive immune microenvironment are rarely confirmed in clinical trial populations. For nanoplatform-based co-delivery, the contrast is particularly pronounced: sophisticated multifunctional nanocarriers routinely achieve >90% tumor growth inhibition in xenograft models, yet none has reached clinical approval [194,195], primarily because GMP-compliant scale-up and consistent EPR-mediated accumulation in human solid tumors remain unsolved challenges. These discrepancies highlight a consistent pattern: preclinical success is necessary but not sufficient for clinical translation. The design of future preclinical studies must more rigorously recapitulate human tumor biology—including hypoxia, immune suppression, and stromal architecture—to narrow the translational gap.

### 7.1. Critical Perspective on Translational Challenges

Despite the promising outlook, the transition of PDT combinations into standard clinical protocols continues to face notable barriers. The primary challenges can be summarized as follows:

1. Dosimetry Inconsistency: In contrast to chemotherapy, where dosing is precisely weight- or body-surface-area-based, PDT efficacy depends on a complex interplay of light fluence, tissue oxygenation, and PS concentration. The lack of real-time, intraoperative dosimetry tools often leads to unpredictable treatment depths and heterogeneous responses [12].
2. Hypoxic Limitations: Most current PDT regimens rely on Type II (oxygen-dependent) photochemical reactions. In the chronically hypoxic TME typical of human malignancies, this mechanism is frequently compromised—an effect often underestimated in well-oxygenated preclinical models [133,197].
3. Regulatory Barriers: Combined “drug-device” modalities encounter a more complex regulatory approval pathway, requiring separate validation of the PS, the light delivery system, and their synergistic interaction [15,198].
4. Preclinical Model Inadequacy: The predominant use of subcutaneous xenograft models in PDT research—which exhibit immunodeficiency and lack stromal complexity and representative tumor vasculature—systematically overestimates efficacy and underestimates hypoxic limitations. Orthotopic syngeneic models, coupled with immune microenvironment characterization, should become the minimum standard for combination PDT studies prior to clinical translation [133,197].
5. Drug-Light Interval (DLI) Standardization: The DLI—the time elapsed between PS administration and light irradiation—governs whether PDT acts primarily through vascular (short DLI: 1–3 h) or cellular (long DLI: 12–48 h) mechanisms, and thus determines the nature of synergy with co-administered agents. Despite its central importance, DLI is rarely reported as a standardized variable in combination studies, making cross-study comparisons unreliable [12]. Mandatory reporting of DLI as a primary protocol parameter in all future combination PDT trials is strongly recommended.

Collectively, these five barriers define a structured translational roadmap: progress will require not only technological innovation but also better preclinical models, consistent reporting, and adaptive regulatory frameworks that can accommodate the complexity of multi-component photodynamic regimens.

Addressing these translational gaps requires a paradigm shift toward personalized, image-guided dosimetry and the development of oxygen-independent (Type I) PSs. Only through such advances can the full preclinical potential of PDT-based combinations be translated into consistent survival benefits for patients.

An ongoing clinical study (NCT05020912) is currently evaluating alterations in the immune microenvironment of basal cell carcinoma following 5-ALA-PDT, including comprehensive assessment of multiple parameters of the antitumor immune response [199]. These and similar mechanistic investigations will be instrumental in guiding the rational design of next-generation PDT-immunotherapy protocols.

### 7.2. Comparative Analysis of Combination Modalities

Taken together, the available preclinical and clinical evidence suggests that the three principal PDT combination strategies differ substantially in their current maturity and optimal clinical context. To facilitate a direct comparison of the available combination strategies and guide clinicians and researchers in the selection of the most appropriate approach in each clinical scenario, Table 7 provides a structured overview of the four principal PDT-based combination paradigms, encompassing their synergistic mechanisms, optimal tumor context, key advantages and limitations, and the current level of clinical evidence.

PDT combined with chemotherapy is supported by the largest body of clinical data and is particularly applicable to anatomically accessible tumors—such as cholangiocarcinoma, endobronchial lung cancer, and cervical cancer—where locoregional PDT can act as a pharmacokinetic primer, enhancing drug accumulation via transient vascular permeabilization. PDT combined with immune checkpoint inhibitors represents the most mechanistically compelling strategy, particularly for tumors with a pre-existing immune infiltrate or those amenable to ICD-mediated immune conversion; this combination is expected to yield the greatest benefit in immunologically ‘cold’ tumors where monotherapy with checkpoint inhibitors is insufficient. PDT combined with targeted agents and photoimmunoconjugates offers the highest degree of tumor selectivity and is most promising for tumors characterized by well-defined receptor overexpression—such as EGFR in head and neck squamous cell carcinoma or triple-negative breast cancer—where receptor-targeted PS delivery ensures precise localization of the photodynamic effect. Across all strategies, four factors serve as the primary determinants of combination selection: (1) tumor oxygenation status—severely hypoxic tumors favor Type I PDT, PACT, or NIR-PIT approaches; (2) immune phenotype—‘cold’ tumors with low TIL density benefit most from PDT + ICI; (3) anatomical accessibility—superficial or endoluminally accessible tumors are better suited to PDT + chemotherapy given current light delivery constraints; and (4) molecular receptor profile—tumors with confirmed EGFR or HER2 overexpression are the optimal candidates for photoimmunoconjugates and NIR-PIT.

### 7.3. Key Unresolved Questions in PDT Combination Therapy

The efficacy of combined PDT in addressing both primary tumors and metastatic spread has been widely validated in various preclinical models. For instance, in orthotopic mouse models of pancreatic ductal adenocarcinoma (PDAC), the combination of verteporfin-PDT and nab-paclitaxel not only suppressed the development of new metastases but also eliminated existing ones in 55% of the subjects. This effect is attributed to treatment-induced biological and physiological alterations within the TME [200]. Similarly, in DMBA-induced breast carcinoma models, combining porphyrin-based PSs with doxorubicin led to enhanced tissue necrosis and the downregulation of key oncogenes such as NRAS, NF-κB, and c-Myc, alongside an up-regulation of the pro-apoptotic caspase-3 gene [201].

Furthermore, advanced tri-modal platforms (chemo-photodynamic-immunotherapy) have demonstrated exceptional results in metastatic settings. In rat models of metastatic melanoma with lung involvement, biomimetic nano-platforms (e.g., PCDD NPs) achieved a 92% inhibition rate for primary tumors and a 90.7% suppression rate for distant metastatic foci [202]. These findings underscore the synergistic potential of PDT to overcome the limitations of conventional therapies in managing systemic malignancy by modulating both local and distant tumor sites.

Despite the growing body of evidence supporting PDT-based combination regimens, several fundamental questions remain unresolved and represent critical barriers to clinical translation. Table 8 provides a systematic analysis of the key unresolved clinical and biological questions that currently preclude the full translation of PDT-based combination regimens into routine oncological practice, detailing for each parameter the current state of knowledge, its mechanistic and clinical significance, and the recommended research priorities for its resolution.

Dosing sequence. The sequence in which PDT and the pharmacological agent are administered has a profound impact on therapeutic outcome, yet no consensus protocol has been established. In the context of PDT + chemotherapy, preclinical data suggest that PDT administered prior to systemic drug delivery is most effective, as it exploits the vascular priming effect to increase intratumoral drug accumulation [117]. However, several studies demonstrate the opposite: pre-treatment with sub-lethal doses of chemotherapy can sensitize tumor cells to subsequent PDT by downregulating antiapoptotic proteins (e.g., Bcl-2) and efflux pumps (e.g., P-glycoprotein) [80,112]. The optimal sequence likely depends on the specific PS–drug combination, tumor type, and treatment intent (debulking vs. sensitization), and requires prospective evaluation in standardized preclinical models before clinical extrapolation.

DLI. The DLI—the time elapsed between PS administration and light irradiation—is a critical but frequently underreported parameter. It determines the intracellular and intravascular distribution of the PS at the moment of activation: shorter DLIs (1–3 h for most second-generation PSs) favor vascular targeting, while longer DLIs (12–24+ hours for porphyrin-based PSs) favor intracellular tumor cell accumulation [12]. In the context of combination therapy, the DLI indirectly governs the dominant cytotoxic mechanism (vascular vs. cellular) and thus the downstream synergy with co-administered agents. For example, vascular-targeted PDT with short DLI creates an ischemic environment that complements subsequent antiangiogenic therapy, whereas cellular PDT with long DLI optimizes ICD induction for synergy with immune checkpoint inhibitors. Standardization of DLI as a reported parameter is essential for meaningful cross-study comparisons and for the rational design of combination protocols.

Oxygen dependence and hypoxia management. The dependence of Type II PDT on molecular oxygen creates an inherent paradox in combination therapy: the same vascular effects that make PDT valuable as a priming agent also exacerbate tumor hypoxia, which can undermine both the photodynamic reaction itself and oxygen-dependent cytotoxic drugs. Current strategies to address this challenge include: (1) the use of Type I PSs (e.g., methylene blue), which generate ROS via electron transfer rather than energy transfer and are therefore effective under hypoxia [52,53]; (2) photoactivated chemotherapy (PACT) with oxygen-independent prodrugs (Pt(IV) complexes) [120]; (3) nanocarrier-mediated oxygen supplementation (perfluorocarbon- or catalase-loaded systems) [51]; and (4) hypoxia-activated combination partners such as tirapazamine. However, none of these strategies has been validated in a clinical randomized setting for combination use, leaving oxygen management as a major unresolved challenge in the design of PDT combination protocols.

Optimal light delivery parameters for combinations. In combination settings, light fluence rate rather than total fluence alone is critical. High fluence rates deplete oxygen faster than it can be replenished, leading to transient hypoxia during irradiation itself, which reduces Type II PDT efficacy and may alter the immunological outcome. Conversely, fractionated or metronomic light delivery protocols can maintain tissue oxygenation and have shown superior efficacy in some preclinical models. The integration of real-time oxygenation monitoring and adaptive light delivery into combination protocols remains an important unresolved technical challenge [12].

Patient selection biomarkers. Across all combination strategies, a critical unresolved question is how to prospectively identify patients most likely to benefit. For PDT + ICI, candidate biomarkers include baseline tumor-infiltrating lymphocyte (TIL) density, PD-L1 expression, and tumor mutational burden. For PDT + chemotherapy, predictors of vascular priming response (tumor vascularity, stromal density) remain undefined. For photoimmunoconjugates, receptor expression levels are a necessary but not sufficient predictor of response. The development and prospective validation of predictive biomarkers is essential for the personalized application of PDT-based combination regimens.

The diverse combination strategies reviewed in this manuscript can be unified within a single conceptual framework. Rather than viewing PDT as a cytotoxic modality used in parallel with other treatments, this framework positions PDT as a biological conditioning agent that operates across three sequential levels to create an optimal environment for downstream pharmacological interventions.

At the physical level, PDT-induced vascular disruption and extracellular matrix degradation reduce interstitial fluid pressure and increase tumor permeability—conditions that maximize the delivery and efficacy of chemotherapy and nanotechnology-based co-delivery [4,117]. At the molecular level, ROS-mediated inactivation of efflux pumps (P-glycoprotein), downregulation of antiapoptotic proteins (Bcl-2), and direct concomitant DNA damage synergize with cytotoxic agents and targeted therapy [6,118]. At the immunological level, PDT-induced ICD and DAMP release prime the immune system, converting immunologically ‘cold’ tumors into ‘hot’ ones—a prerequisite for synergy with immune checkpoint inhibitors and antitumor vaccines [7,8,26]. Nanotechnology serves as the delivery infrastructure that simultaneously exploits all three levels by co-delivering PSs and pharmacological agents in a synchronized, stimulus-responsive manner.

This explains why different combination strategies are optimal under different tumor-specific biological conditions: hypoxic tumors favor Type I PSs and PACT; immunologically cold tumors favour PDT + ICI; tumors with dense stroma favour PDT as a vascular primer before chemotherapy; and tumors with well-defined receptor expression favor photoimmunoconjugates. Critically, hypoxia and immune modulation are not isolated challenges but cross-cutting variables that influence the efficacy of every combination strategy.

## 8. Conclusions

Photodynamic therapy is rapidly evolving from a localized cytotoxic modality into a multifunctional platform for TME reprogramming, precision drug delivery, and immune activation. As demonstrated throughout this review, the greatest translational potential of PDT lies not in its use as a standalone treatment, but in its rational integration with chemotherapy, immunotherapy, targeted agents, and smart nanotechnology-based delivery systems.

The therapeutic superiority of these combinations is driven by several complementary mechanisms, including vascular priming, the reversal of multidrug resistance, induction of immunogenic cell death, stromal remodeling, and controlled spatiotemporal drug release. Importantly, the optimal combination strategy should be selected according to tumor-specific biological parameters, including oxygenation status, immune phenotype, stromal density, anatomical accessibility, and receptor expression.

However, this field must also recognize critical limitations. Clinical and preclinical failures most often arise from inadequate dosimetry, poor control of the drug–light interval, overreliance on inconsistent EPR effects, neglect of tumor hypoxia, excessive nanoplatform complexity, and the use of superficial irradiation strategies for bulky or deeply seated lesions. Future progress depends not only on developing more sophisticated platforms, but also on avoiding biologically and technically predictable design errors.

The next stage of clinical translation should prioritize simplified and scalable multifunctional systems, standardized light-delivery protocols, real-time dosimetry, robust patient stratification, and mechanism-driven sequencing of drug and light exposure. Addressing these unresolved issues is essential for transforming PDT-based combination therapy from a highly promising experimental strategy into a reproducible component of precision oncology.

Based on the synthesis presented in this review, we propose the following five research priorities for the next phase of PDT combination therapy development: (1) adoption of orthotopic syngeneic tumor models with immune microenvironment profiling as the minimum preclinical standard; (2) mandatory standardization of DLI reporting and light fluence parameters in all combination PDT clinical trials, modeled on established dosimetry frameworks; (3) clinical validation of Type I PSs and PACT approaches in hypoxic tumor subtypes (glioblastoma, pancreatic cancer) where Type II PDT has consistently failed to translate; (4) development of validated predictive biomarkers—including baseline TIL density, circulating DAMP levels, and PS tumor uptake quantified by fluorescence imaging—for prospective patient stratification in PDT + ICI trials; and (5) engagement with regulatory bodies for pre-IND meetings to develop adaptive approval pathways for combination PDT drug-device products. Progress on these five fronts will determine whether PDT-based combinations achieve their substantial preclinical potential in the clinical setting.

## Figures and Tables

**Table 2:** In vitro and in vivo studies investigating the combination of PDT and cytostatics.

Photosensitizer	Cytostatic	Tumor/Model/Effect	Reference
MB	Rutoside	A375 lung cancer cell line; induction of apoptosis and cell cycle arrest in tumor cells	[92]
AlPc (Photosens^®^)	Doxorubicin, Methotrexate	HeLa cell lines (cervical cancer), MCF-7 (breast adenocarcinoma), and RG2 (rat glioblastoma); cytotoxic effect most pronounced when chemotherapy preceded PDT by 24 h	[93]
ZnPc	Doxorubicin	SK-MEL-3 (human melanoma cells); induction of apoptosis via a caspase-dependent pathway (caspase-8, -9, and -3 expression) and reduced cell migration capacity	[94]
Hp	Cisplatin	A549 lung adenocarcinoma in BALB/c-nu/nu mice; tumor growth inhibition of 67.1% in the combination group and an increase in the apoptosis index to 9.5% via modulation of the PI3K/AKT signaling pathway	[95]
5-ALA	Doxorubicin, Carboplatin	Various carcinoma types (adenocarcinomas, fibrosarcomas, transitional cell carcinoma, and others) in dogs (dachshunds, retrievers, mongrels, chihuahuas, and others); long-term tumor control was observed. A correlation was identified between the expression of mRNA involved in 5-ALA-induced PpIX accumulation and PpIX concentration in canine tumor cells	[96]

Abbreviations: MB—Methylene blue; AlPc—Aluminum phthalocyanine; ZnPc—Zinc phthalocyanine; Hp—Hematoporphyrin; 5-ALA—5-aminolevulinic acid; PDT—Photodynamic therapy, PpIX—Protoporphyrin IX.

**Table 3:** Clinical application of PDT and cytostatics in the treatment of human tumors.

Photosensitizer	Cytostatic	Tumor/Effect	Reference
Porfimer sodium (Photofrin^®^)	Gemcitabine/Cisplatin	16 patients with advanced cholangiocarcinoma; increased survival in the combined therapy group	[99]
PSD-007 (Photocarcinorin)	5-fluorouracil	140 patients with advanced esophagocardiac cancer, the rate of remission in the PDT + CT group was significantly higher (*p* < 0.05), the mean survival time was longer than that of the PDT group (*p* < 0.01).	[104]
Ce6	Paclitaxel and platinum-based agents	21 patients with stage IIIA–IIIB non-small cell lung cancer. Objective response rate—90% in the combined therapy group vs. 76% in the No-PDT group; R0 resection rate—89% in the PDT group vs. 54% in the No-PDT group	[106]

Abbreviations: Ce6—chlorin e6; PDT—photodynamic therapy; CT—chemotherapy.

**Table 4:** Experimental studies investigating the combination of immune checkpoint inhibitors with PDT.

Photosensitizer	Immuno-Oncology Drug	Tumor/Effect	Reference
EITC	Anti-PD-L1 antibody (atezolizumab)	Non-small cell lung cancer cell lines (H1975—PD-L1-high and A549—PD-L1-low); enhanced cytotoxicity in PD-L1-high H1975 cells; cell-specific intracellular transport of atezolizumab	[156]
UCNP-Ce6-R837	Anti-PD-L1 antibodies	CT26 cells (murine colon carcinoma); BALB/c mice with bilateral subcutaneous CT26 cell implantation (colorectal cancer model); cytotoxic effect; delivery of anti-PD-L1 antibody to the tumor; inhibition of both irradiated and non-irradiated (abscopal) tumor growth; formation of immunological memory—animals with complete tumor regression rejected CT26 cell re-challenge	[157]
Bremachlorin	Anti-PD-1 antibodies	Pancreatic ductal adenocarcinoma; mice; increased survival; enhanced sensitivity to immune checkpoint inhibitors. One mouse with tumors characterized by high T cell infiltration exhibited complete regression of both irradiated and non-irradiated distant tumors 90 days post-treatment	[158]
HpD	Anti-PD-1 antibodies	4T1 metastatic breast cancer model; bilateral subcutaneous transplantation; BALB/c mice; tumor growth inhibition; increased animal lifespan; immune response modulation	[159]
Ce6 with ICG, NATNgel nanoplatform	Anti-PD-1 antibodies	Lung and colon cancer models; subcutaneous implantation; mice; complete regression of rapidly growing tumors; complete suppression of re-implanted tumor growth; minimal damage to healthy tissues	[160]

Abbreviations: EITC—Eosin-5-isothiocyanate; HpD—hematoporphyrin derivatives; PD-L1—programmed death-ligand 1; UCNP-Ce6-R837—chlorin e6 (Ce6) and imiquimod (R837) coloaded upconversion nanoparticles (UCNPs); ICG—Indocyanine green; NATNgel—near-infrared (NIR) fluorescent dye-loaded activatable theranostic nanogel.

**Table 5:** Clinical trials investigating the combination of PDT and immune checkpoint inhibitors.

Photosensitizer	ICI Agent	Tumor Type	Clinical Trial Number	Reference
Porfimer sodium	Nivolumab/ Pembrolizumab	Locally advanced or metastatic head and neck cancer	NCT03727061	[163]
5-ALA	Nivolumab	Malignant pleural mesothelioma	NCT04400539	[164]

Abbreviations: ICI—Immune checkpoint inhibitors; 5-ALA—5-aminolevulinic acid.

**Table 7:** Comparative analysis of PDT-based combination strategies: mechanisms, clinical context, advantages, limitations, and unresolved questions.

Combination Strategy	Synergistic Mechanism	Optimal Tumor Context	Key Advantages	Current Limitations	Key Unresolved Questions	Evidence Level
PDT + Chemotherapy	(1) Vascular priming: transient ↑ permeability and ↓ interstitial fluid pressure (IFP) → 3–5× ↑ drug accumulation [117]; (2) inactivation of efflux pumps (P-glycoprotein) → restoration of drug sensitivity [6]; (3) complementary cell death induction via ROS + DNA damage [80]	Anatomically accessible solid tumors; desmoplastic and chemoresistant disease (cholangiocarcinoma, NSCLC, cervical cancer); tumors with high stromal density where drug penetration is limited	Dose de-escalation of cytotoxics; synergistic apoptosis induction; largest body of clinical evidence among PDT combinations	Efficacy strictly oxygen-dependent; PDT-induced hypoxia can paradoxically impair subsequent drug delivery; limited RCT data; endpoints inconsistent across studies	Optimal administration sequence (PDT before vs. after chemotherapy); standardization of drug-light interval (DLI); impact of PDT-induced hypoxia on drug PK	Moderate. Prospective trials ongoing (NCT02082522, NCT04099888, NCT05736406) [100,101,107]; retrospective cohort studies with consistent direction of effect [97,98,103]
PDT + Immunotherapy (ICIs, DC vaccines)	(1) ICD induction → release of DAMPs (calreticulin, HMGB1, ATP) → DC maturation and T-cell priming [7,8,26]; (2) “cold-to-hot” tumor immune conversion [153]; (3) PDT-mediated PD-L1 upregulation → increased ICI response probability [153]; (4) in situ or ex vivo DC vaccination using PDT-treated tumor cell lysates [175,180]	Immunologically “cold” tumors with low baseline TIL density; metastatic disease with distant micrometastases; tumors refractory to ICI monotherapy	Abscopal effect (suppression of non-irradiated lesions) [157,158]; potential for durable systemic immunity; synergy with ICI restores T-cell infiltration; vaccine approach enables personalization	Immunosuppressive rebound (Treg expansion, MDSC recruitment) limits response durability [26,125]; efficacy highly dependent on patient immune status; no standardized antigen harvesting protocol	Optimal timing of ICI relative to PDT (before/simultaneously/after); predictive biomarkers for patient selection (TIL density, PD-L1, TMB); optimal protocol for DC vaccine preparation	Low-to-Moderate. Phase I/II trials ongoing (NCT03727061, NCT04400539) [163,164]; robust preclinical evidence [153,154,155,156,157,158,159,160]
PDT + Targeted Therapy/Photoimmunoconjugates (NIR-PIT)	(1) Receptor-targeted PS delivery (anti-EGFR, anti-HER2) → highly selective photodamage [165]; (2) NIR-PIT: IR700 antibody conjugates → ligand-induced membrane disruption independent of oxygen [167,168]; (3) TKI co-delivery → vascular normalization → ↑ tumor oxygenation and improved PDT efficacy [186]; (4) enzyme-responsive dual-action conjugates (PS + TKI) [166]	Tumors with confirmed receptor overexpression (EGFR in HNSCC, TNBC; HER2 in breast cancer); tumors adjacent to critical anatomical structures (head and neck); hypoxic tumor regions (NIR-PIT)	Highest tumor selectivity among PDT combinations; oxygen-independent cell killing (NIR-PIT effective in hypoxia); dual mechanism of action (photodamage + signaling inhibition)	Requires well-defined and homogeneous receptor expression—limited to specific tumor subtypes; complex manufacturing and quality control of conjugates; receptor downregulation can abolish activity	Management of intratumoral receptor heterogeneity; optimization of antibody-to-PS ratio in conjugates; combination with systemic vs. local light delivery	Low. Phase III ECLIPSE trial (NCT06699212) initiated 2024; most evidence from phase I/II studies and preclinical models [165,166,167,168,172,186]
Nanotechnology-Based Co-delivery	(1) Synchronized pharmacokinetics of PS + drug at fixed molar ratios within a single nanocarrier; (2) stimuli-responsive release (pH, GSH, light, enzymatic triggers) → tumor-selective activation [166,186]; (3) EPR effect + active targeting (antibody, peptide, folic acid) → ↑ intratumoral accumulation [6,165]; (4) sub-therapeutic PDT as “vascular priming” to further enhance nanoparticle penetration [117]	Tumors with functional EPR effect; high-interstitial-pressure desmoplastic tumors (pancreatic, breast) where stromal preconditioning by PDT improves penetration; cases requiring dose reduction in toxic agents (e.g., cardiotoxic anthracyclines)	Simultaneous delivery of multiple therapeutic modalities; reduced systemic toxicity (e.g., cardiotoxicity of DOX reduced with PDA-liposomes [187]); single light trigger activates both PDT and drug release; scalable to include targeting moieties	High synthetic complexity limits GMP-compliant scale-up [194,195]; EPR effect heterogeneous and unreliable in desmoplastic tumors [112]; long-term safety of non-biodegradable components unclear [196]; complex “drug-device” regulatory pathway [15,198]	Scalable and reproducible GMP-compliant manufacturing; standardization of particle characterization for regulatory submission; EPR effect optimization in human tumors	Preclinical. No approved combination nanoplatform PDT regimens; selected liposomal PSs in early-phase clinical evaluation

Abbreviations: ICD—immunogenic cell death; DAMPs—damage-associated molecular patterns; ICI—immune checkpoint inhibitor; DC—dendritic cell; TIL—tumor-infiltrating lymphocytes; TMB—tumor mutational burden; MDSC—myeloid-derived suppressor cell; IFP—interstitial fluid pressure; PS—photosensitizer; TKI—tyrosine kinase inhibitor; NIR-PIT—near-infrared photoimmunotherapy; EPR—enhanced permeability and retention; GMP—Good Manufacturing Practice; GSH—glutathione; PK—pharmacokinetics; NSCLC—non-small cell lung cancer; HNSCC—head and neck squamous cell carcinoma; TNBC—triple-negative breast cancer; DOX—doxorubicin; DLI—drug-light interval. Note on symbols: ↑—increase/upregulation; ↓—decrease/downregulation; →—leads to/results in.

**Table 8:** Key unresolved clinical and biological questions in PDT-based combination therapy: current status and research priorities.

Combination	Unresolved Parameter	Current Status of Knowledge	Why It Matters for Translation	Recommended Research Direction
PDT + Chemotherapy	Administration sequence (PDT before vs. after chemo)	PDT-first exploits vascular priming and ↑ drug accumulation 3–5× [117]; chemo-first can downregulate efflux pumps and antiapoptotic proteins [80,112]. No head-to-head clinical comparison exists	Sequence determines the dominant synergistic mechanism and may be tumor-type specific	Prospective randomized crossover studies in defined tumor models; preclinical PK/PD modeling of sequence-dependent effects
PDT + Chemotherapy	Drug-light interval (DLI)	Short DLI (1–3 h) → vascular PS distribution → vascular targeting; long DLI (12–48 h) → intracellular PS distribution → direct tumor cell PDT [12]. Interaction between DLI and optimal chemotherapy timing is unexplored	DLI governs the dominant cytotoxic mechanism and thus the nature of synergy with co-administered drugs	Standardized reporting of DLI in all preclinical and clinical combination studies; dedicated dose-escalation studies testing DLI as an independent variable
PDT + Chemotherapy	Impact of PDT-induced hypoxia on drug PK	PDT-induced vascular shutdown creates acute hypoxia that may reduce oxygen-dependent drug efficacy and alter drug metabolism. The net pharmacokinetic effect of this hypoxic window on co-administered drugs is poorly characterized	Acute hypoxia after PDT could negate the vascular priming benefit if chemotherapy is delivered too late after the hypoxic peak	Real-time oxygen tension monitoring combined with pharmacokinetic sampling; optimization of the post-PDT window for drug delivery
PDT + Immunotherapy (ICIs)	Timing of ICI relative to PDT	ICI before PDT may normalize the TME and enhance T-cell infiltration prior to ICD induction; ICI after PDT exploits the immunogenic window created by DAMPs. No clinical data directly compare these sequences	Incorrect timing may result in T-cell exhaustion before ICD is triggered, or ICI activity may be wasted before the immune-priming effect of PDT is established	Phase II trials with randomized arms testing PDT → ICI vs. concurrent vs. ICI → PDT sequences; translational immunomonitoring of DAMP release kinetics
PDT + Immunotherapy (ICIs)	Predictive biomarkers for patient selection	Candidate biomarkers proposed include TIL density, PD-L1 expression, TMB, and DAMPs (calreticulin, HMGB1 serum levels); none prospectively validated for PDT + ICI combinations [153]	Without validated biomarkers, patient selection is empirical, leading to heterogeneous trial populations and inconsistent results	Biomarker-enriched basket trials; multiplex immunohistochemistry of pre-treatment biopsies; circulating DAMP profiling as a pharmacodynamic readout
PDT + Immunotherapy (DC vaccines)	Optimal timing and protocol for ICD-based antigen harvesting	PDT conditions that highly induce ICD (PS concentration, light dose, DLI) differ from those that maximize direct cytotoxicity; the optimal harvest point is undefined. Freeze–thaw lysate vs. whole irradiated cells as vaccine substrate also remains unresolved [181,182,183,184]	Protocol variability is the primary cause of inconsistent immunogenicity across DC vaccine studies	Systematic in vitro screening of PDT conditions for DAMP release; standardized protocols for DC loading and maturation; reporting of calreticulin surface exposure as a quality metric
PDT + Targeted Therapy/NIR-PIT	Intratumoral receptor heterogeneity	Receptor overexpression (EGFR, HER2) is measured from biopsy samples that may not reflect the full intratumoral distribution; receptor downregulation under therapeutic pressure can abolish conjugate binding and activity [165]	Even a minor receptor-negative subpopulation can escape photoimmunoconjugate-mediated killing and serve as the source of recurrence	Multiplex imaging of receptor distribution in pre-treatment surgical or biopsy specimens; combination with receptor-independent PDT for heterogeneous tumors
PDT + Targeted Therapy/NIR-PIT	Antibody-to-PS ratio and conjugate stability	The IR700 dye-to-antibody ratio critically influences conjugate hydrophobicity, aggregation, and pharmacokinetics; no consensus ratio has been validated across tumor types [167,168]	Suboptimal conjugate stability leads to premature PS release, off-target phototoxicity, and reduced tumor accumulation	Systematic conjugate optimization studies with standardized characterization panels; development of in silico pharmacokinetic models for conjugate biodistribution
Nanotechnology-Based Co-delivery	EPR effect reliability in human tumors	The EPR effect, which underlies passive nanoparticle accumulation, is robust in murine models but heterogeneous and often insufficient in human desmoplastic tumors (pancreatic, breast). Sub-therapeutic PDT preconditioning can improve penetration 3–5× [117] but has not been validated clinically	If the EPR effect is unreliable in the target tumor type, passive nanoparticle accumulation fails, and the rationale for co-delivery platforms is undermined	Clinical pharmacokinetic studies of PS-drug nanoplatforms with tumor biopsy sampling; development of imaging biomarkers (e.g., [^64^Cu]-labeled nanoparticles) to pre-screen EPR functionality
Nanotechnology-Based Co-delivery	GMP-compliant scale-up and batch reproducibility	Most published nanoplatforms are synthesized in 3–5 mg quantities under academic laboratory conditions; transition to GMP-compliant production at the >100 g scale introduces formulation instability, reduced encapsulation efficiency, and batch variability [194,195]	Scale-up failure is the most common reason for discontinuation of otherwise promising nanomedicines between preclinical and phase I stages	Early engagement with regulatory bodies (FDA, EMA) for pre-IND meetings; adoption of quality-by-design (QbD) frameworks; development of continuous manufacturing processes
All combinations	Fluence rate and oxygen dynamics during PDT	High fluence rate depletes oxygen faster than tissue replenishment → transient intraprocedural hypoxia → reduced Type II PDT efficiency. Metronomic (low fluence rate, prolonged) delivery maintains oxygenation but prolongs procedure time [12]	In combination protocols, fluence rate determines whether PDT acts as a cytotoxic or immunogenic primer—highrate favors necrosis, low-rate favors ICD and apoptosis	Real-time intraoperative oxygenation monitoring (diffuse optical spectroscopy); prospective comparison of fractionated vs. continuous light delivery in combination trials
All combinations	Long-term immunological memory	Preclinical models show that successful PDT + ICI combinations generate immunological memory that rejects tumor rechallenge [157,158]. Whether this translates to durable clinical responses and how to measure it remain undefined	Immunological memory is the ultimate goal of combination immunotherapy—its absence indicates that only short-term tumor control, not cure, has been achieved	Long-term follow-up arms in all PDT + ICI trials; integration of memory T-cell phenotyping (T~SCM~, T~CM~) into correlative studies

Abbreviations: DLI—drug-light interval; PK—pharmacokinetics; PD—pharmacodynamics; ICI—immune checkpoint inhibitor; ICD—immunogenic cell death; DAMPs—damage-associated molecular patterns; TIL—tumor-infiltrating lymphocytes; TMB—tumor mutational burden; DC—dendritic cell; TME—tumor microenvironment; PS—photosensitizer; NIR-PIT—near-infrared photoimmunotherapy; EGFR—epidermal growth factor receptor; EPR—enhanced permeability and retention; GMP—Good Manufacturing Practice; QbD—quality by design; T~SCM~—stem cell memory T cells; T~CM~—central memory T cells. Note on symbols: ↑—increase/upregulation; →—leads to/results in.

## Data Availability

No new data were created or analyzed in this study. Data sharing is not applicable to this article.

## Abbreviations

The following abbreviations are used in this manuscript:

PDT	Photodynamic therapy
TME	Tumor microenvironment
ICD	Immunogenic cell death
PS	Photosensitizer
ROS	Reactive oxygen species
EPR	Enhanced permeability and retention
DAMPs	Damage-associated molecular patterns
ICIs	Immune checkpoint inhibitors
DC	Dendritic cell
NIR-PIT	Near-infrared photoimmunotherapy
COX-2	Cyclooxygenase-2
PACT	Photoactivated chemotherapy
HpD	Hematoporphyrin derivatives
Ce6	Chlorin e6
HPPH	2-[1-hexyloxyethyl]-2-devinyl pyro pheophorbide-a
NIR	Near-infrared
ICG	Indocyanine green
HCC	Hepatocellular carcinoma
PPa	Pheophorbide-a
ZnPc	Zinc phthalocyanine
VTP	Vascular-targeted photodynamic
NPs	Nanoparticles
LDL	Low-density lipoprotein
iPDT	Interstitial PDT
DNA	Deoxyribonucleic acid
BC	Breast cancer
MB	Methylene blue
DOX	Doxorubicin
5-ALA/ALA	5-aminolevulinic acid
SCLC	Small-cell lung cancer
AlPc	Aluminum phthalocyanine
Hp	Hematoporphyrin
PpIX	Protoporphyrin IX
PI3K/AKT	Phosphoinositide 3-kinase/Protein kinase B signaling pathway
PCI	Photochemical internalization
NSCLC	Non-small cell lung cancer
ECOG	Eastern Cooperative Oncology Group
ECA	External carotid artery
CT	Computed tomography (or Chemotherapy in some contexts)
mFOLFIRINOX	Modified FOLFIRINOX (5-fluorouracil, leucovorin, irinotecan, and oxaliplatin)
CSCs	Cancer stem cells
OXPHOS	Oxidative phosphorylation
PDP	Photodynamic priming
ECM	Extracellular matrix
IFP	Interstitial fluid pressure
GSH	Glutathione
MDSC	Myeloid-derived suppressor cell
PGE2	Prostaglandin E2
NSAIDs	Non-steroidal anti-inflammatory drugs
VEGF	Vascular Endothelial Growth Factor
AIE	Aggregation-induced emission
TAAs	Tumor-associated antigens
ATP	Adenosine triphosphate
CRT	Calreticulin
CTL	Cytotoxic T lymphocyte
CTLA-4	Cytotoxic T-lymphocyte-associated protein 4
EITC	Eosin-5-isothiocyanate
UCNPs	Upconversion nanoparticles
UCNP-Ce6-R837	Ce6 and imiquimod (R837) co-loaded upconversion nanoparticles (UCNPs)
NATNgel	NIR fluorescent dye-loaded activatable theranostic nanogel
TNF-α	Tumor necrosis factor-alpha
PD-L1	Programmed death-ligand 1
HIF-1α	Hypoxia-inducible factor 1-alpha
EGFR	Epidermal growth factor receptor
HER2	Human epidermal growth factor receptor 2
HNSCC	Head and neck squamous cell carcinomas
PIT	Photoimmunotherapy
CPS	Combined Positive Score
PDT-DC	Photodynamically sensitized dendritic cell
ER	Endoplasmic reticulum
PNPs	Polymeric nanoparticles
NLCs	Nanostructured lipid carriers
AuNPs	Gold nanoparticles
PDA	Polydopamine
pNALs	Photoactivable nanoliposomes
Combo-NP	Biodegradable fluorescent NIR-II pseudoconjugated polymer nanoparticle
NIR-II	Near-infrared II
CA4P	Combretastatin A4 phosphate
ICG-Lipo-C&D	Liposomally formulated indocyanine green derivative (ICG-Lipo) encapsulating carboplatin and docetaxel (C&D)
GA/RGD-DOX/ICG-Lips	Glycyrrhetinic acid (GA)/arginine-glycine-aspartate (RGD)-targeted and doxorubicin (DOX)/indocyanine green (ICG)-loaded Liposomes
GMP	Good Manufacturing Practice
DLI	Drug-Light Interval
TIL	Tumor-infiltrating lymphocytes
TMB	Tumor mutational burden
TKI	Tyrosine kinase inhibitor
PK	Pharmacokinetics
TNBC	Triple-negative breast cancer
PDAC	Pancreatic ductal adenocarcinoma
DMBA	7,12-dimethylbenz[a] anthracene
PCDD NPs	Platelet membrane-coated nanoparticles
QbD	Quality by design
